# Synthesis, Computational Pharmacokinetics Report, Conceptual DFT-Based Calculations and Anti-Acetylcholinesterase Activity of Hydroxyapatite Nanoparticles Derived From *Acorus Calamus* Plant Extract

**DOI:** 10.3389/fchem.2021.741037

**Published:** 2021-10-07

**Authors:** Sushma Pradeep, Anisha S. Jain, Chandan Dharmashekara, Shashanka K. Prasad, Nagaraju Akshatha, R. Pruthvish, Raghavendra G Amachawadi, Chandrashekar Srinivasa, Asad Syed, Abdallah M. Elgorban, Abdulaziz A. Al Kheraif, Joaquín Ortega-Castro, Juan Frau, Norma Flores-Holguín, Chandan Shivamallu, Shiva Prasad Kollur, Daniel Glossman-Mitnik

**Affiliations:** ^1^ Department of Biotechnology and Bioinformatics, School of Life Sciences, JSS Academy of Higher Education and Research, Mysuru, India; ^2^ Department of Physics, Marimallapa PU College, Mysuru, India; ^3^ Department of Biotechnology, Acharya Institute of Technology, Bengaluru, India; ^4^ Department of Clinical Sciences, College of Veterinary Medicine, Kansas State University, Manhattan, KS, United States; ^5^ Department of Studies in Biotechnology, Davangere University, Shivagangothri, Davangere, India; ^6^ Department of Botany and Microbiology, College of Science, King Saud University, Riyadh, Saudi Arabia; ^7^ Dental Health Department, College of Applied Medical Sciences, King Saud University, Riyadh, Saudi Arabia; ^8^ Departament de Química, Universitat de les Illes Balears, Palma de Mallorca, Spain; ^9^ Laboratorio Virtual NANOCOSMOS, Departamento de Medio Ambiente y Energía, Centro de Investigación en Materiales Avanzados, Chihuahua, México; ^10^ Department of Sciences, Amrita School of Arts and Sciences, Amrita Vishwa Vidyapeetham, Mysuru Campus, Mysuru, India

**Keywords:** Alzheimer’s disease, neuropathology, neurofibrillary tangles, amyloid plaques, molecular docking, computational pharmacokinetics, Conceptual DFT

## Abstract

Over the years, Alzheimer’s disease (AD) treatments have been a major focus, culminating in the identification of promising therapeutic targets. A herbal therapy approach has been required by the demand of AD stage-dependent optimal settings. Present study describes the evaluation of anti-acetylcholinesterase (AChE) activity of hydroxyapatite nanoparticles derived from an *Acorus calamus* rhizome extract (AC-HAp NPs). The structure and morphology of as-prepared (AC-HAp NPs) was confirmed using powder X-ray diffractometer (XRD), scanning electron microscopy (SEM), transmission electron microscopy (TEM) and high-resolution transmission electron microscopy (HR-TEM). The crystalline nature of as-prepared AC-HAp NPs was evident from XRD pattern. The SEM analysis suggested the spherical nature of the synthesized material with an average diameter between 30 and 50 nm. Further, the TEM and HR-TEM images revealed the shape and size of as-prepared (AC-HAp NPs). The interplanar distance between two lattice fringes was found to be 0.342 nm, which further supported the crystalline nature of the material synthesized. The anti-acetylcholinesterase activity of AC-HAp NPs was greater as compared to that of pure HAp NPs. The mechanistic evaluation of such an activity carried out using in silico studies suggested that the anti-acetylcholinesterase activity of phytoconstituents derived from *Acorus calamus* rhizome extract was mediated by BNDF, APOE4, PKC-*γ*, BACE1 and *γ*-secretase proteins. The global and local descriptors, which are the underpinnings of Conceptual Density Functional Theory (CDFT), have been predicted through the MN12SX/Def2TZVP/H2O model chemistry to help in the comprehension of the chemical reactivity properties of the five ligands considered in this study. With the further objective of analyzing their bioactivity, the CDFT studies are complemented with the estimation of some useful computed pharmacokinetics indices, their predicted biological targets, and the ADMET parameters related to the bioavailability of the five ligands are also reported.

## 1 Introduction

The most frequent form of dementia is Alzheimer’s disease. It affects millions of individuals around the world, and the number is rapidly increasing. Alzheimer’s disease has been found to impact socially and financially the lives of those affected (Wang et al. 2016). According to the amyloid hypothesis, misfolding of the extracellular protein collected in senile plaques and intracellular deposition of misfolded tau protein in neurofibrillary tangles induce memory loss and disorientation, as well as personality and cognitive decline over time (Förstl and Kurz 1999). Over 24 million people worldwide are estimated to have dementia, with Alzheimer’s disease accounting for the bulk of cases (Mayeux and Stern 2012). As a result, research into Alzheimer’s disease, which is a huge public health concern, must be prioritized. The treatments available are aimed at alleviating the symptoms of Alzheimer’s disease, implying the necessity to have a better understanding of disease pathophysiology to find/develop treatments that can lessen symptoms or repair harm already done (Solfrizzi et al. 2011). The most important component in focusing therapy efforts is integrating both pharmaceutical and psychosocial support systems towards early diagnosis and studying the disease further.

The absence of treatment for Alzheimer’s disease and dementia has become a major public health concern. Alzheimer’s disease is a neurological disease that worsens with time Heneka et al. (2015). The therapeutic drugs used to treat Alzheimer’s disease must either cure or slow the illness. There are few first-line medications/drugs available to treat AD and these works as acetylcholinesterase inhibitors and have FDA approval in the United States Wilson et al. (2011). However, because none of the treatments are designed to boost neural functioning, none of them can entirely heal the disease or improve the patient’s cognitive or memory skills. As a result, there is a need for an alternative supply of medication to treat Alzheimer’s disease Blennow et al. (2006). The amyloid hypothesis, presented in 1991, claimed that AD was caused by the accumulation of Aβ proteins. The APP (amyloid *β*-protien precursor) gene, which produces Aβ protein, is found on the 21st chromosome, and people with Down’s syndrome (trisomy 21) have an extra copy of this gene, resulting in the earliest reported symptom(s) of Alzheimer’s disease at the age of 40 Waring and Rosenberg (2008). APOE4 (Apolipoprotein E4) has long been thought to be a key risk factor for Alzheimer’s disease since it aids in the breakdown of Aβ proteins. However, some isoforms of APOE4 are ineffective, resulting in amyloid buildup in the brain Selkoe (1999). Also, the enzyme acetylcholinesterase is involved in cholinergic neurotransmission. It degrades acetylcholine stopping the neurotransmission process. The assay of AChE activity can be used to confirm the efficacy of various test substances as herbal extracts or herbal extracts derived nanoparticles in terms of treatment Kim (2018).

Ayurveda is an ancient medicinal system that employs a variety of herbs and plants to effectively treat a wide range of diseases Strittmatter et al. (1993). Herbal treatments from plants contain a blend of phytocompounds with varying pharmaco-biological relevance and can treat a variety of disorders. Plants, in fact, have long been a primary source of medications in a variety of therapeutic traditions Mahley et al. (2006). The plant’s pharmacological activity, such as anti-amyloidogenic, anti-inflammatory, antioxidants and anti-cholinesterase properties, are due to phytochemical components such as polyphenols, alkaloids, triterpenes, tannins, lignins, sterols and flavonoids Francis et al. (1999). *Acorus calamus*, a member of the *Acoraceae* family, is native to India. In Ayurveda, this plant is revered for its revitalizing effects on the neurological system, brain, and digestive systems. Alkaloids, volatile oil, steroids, tannins, sesquiterpenes, polyphenols, saponin, lignin, mucilage, monoterpenes, flavonoids and glycosides chemicals are among the phytoconstituents found in Ayurveda Martorana et al. (2010). *A. calamus* has anti-microbial, anti-ulcer, antidiabetic, insecticidal, neuroprotective, anti-allergic, anti-inflammatory, cardioprotective, pesticidal, anti-cancer and anti-oxidant activities. Ferreira-Vieira et al. (2016).

Nanomedicines (NMs) have a number of unique qualities that allow them to deliver anti-AD therapies to specific brain locations Spuch et al. (2012). NMs benefit from smaller dimensions and enhanced biocompatibility, making therapeutic chemicals easier to move into the brain Fakhoury et al. (2015). NMs that are small (about 100–10,000 times smaller than a human cell) can easily interact with proteins and chemicals on the cell surface and inside the cell. The essential core structures of NP-functionalized NMs ensure drug encapsulation or conjugation, as well as protection and sustained blood circulation Leszek et al. (2017). NMs can also target cells or even an intracellular compartment such as Aβ in cells, allowing the drug to be delivered at a predetermined dosage straight to the diseased spot Kim et al. (2012). NMs can reduce the dose and frequency of treatment, resulting in better patient compliance Altinoglu and Adali (2020). Nanomedicines have potential advantages over other conventional ways of drug delivery to the brain to cure AD, such as favorability to the brain, greater stability, biocompatibility and biodegradability, protection from enzymatic degradation, increased half-life, improved bioavailability, and controlled release, despite some clinical issues Knop et al. (2010).

Hydroxyapatite (Ca_10_(PO_4_)_6_(OH)_2_) is composed of 70% apatite calcium phosphate and remaining 30% of natural materials Gopi et al. (2013). As a result, it is frequently employed in biomedical applications including fillers for bone deformities, scaffolds for tissue engineering, coatings on metallic implants to increase biocompatibility, and drug/protein delivery carriers Youness et al. (2017). On the other hand, green synthesis of nanoparticles using plant leaf extracts has opened a new era in research.

A quite often method of predicting a small molecule’s orientation when it is bound to a target molecule to create a stable complex is the *in silico* molecular docking approach Davies (1999). Predicting the strength of association or binding affinity between two molecules requires knowledge of the preferred orientation. The study of how two or more molecular structures fit together is known as molecular docking. As a result, molecular docking can be used to forecast the strength that will be created between the molecules Polvikoski et al. (1995). The binding behavioral studies have aided in understanding the fundamental biological processes that help in rational drug discovery techniques Lacor et al. (2007). Rational drug discovery (RDD) study was to find an inhibitor that binds and stops the action of some toxic proteins produced in the human body. RDD allows researchers to forecast how tiny molecules like ligands bind in the receptor target site. One of the most often used strategies in structure-based drug design is molecular docking Nikolaev et al. (2009). The present study investigates the *in silico* and AChE inhibitory activity of pure HApNPs and Ac-HApNPs prepared using aqueous extract of rhizome of *Acorus calamus* against AD proteins.

## 2 Materials and Methods

All the chemicals and reagents were procured from Loba chemicals (Bangalore, India). Demineralized water was collected from an ELGA RO system and was used throughout the experiments (Elga Veolia, Lane End, United Kingdom). The crystalline phases were recorded on Bruker X-ray diffractometer with a scan range of 20–70° at a 2°/min scan rate using Cu Kα (1.5406 Å) radiation (Bruker, Karlsruhe, Germany). The morphology and elemental composition were studied using Scanning electron microscopy (SEM) and Energy dispersive X-ray (EDX) mapping, respectively, which were recorded on a Zeiss microscope (Carl Zeiss, White Plains, NY, United States). Transmission electron microscopy (TEM) images and Selected Area Electron Diffraction (SAED) patterns were recorded on a JEOL 2100F FEG apparatus operating at 200 kV after casting a drop of sample material for dispersion in ethanol over a Cu grid (JEOL, Akishima, Tokyo, Japan).

### 2.1 Plant Material Collection

The matured rhizomes of the *Acorus calamus* plant grown were collected in the region around Mysuru, Karnataka, India. The rhizomes collected were washed with single distilled water and 0.5% sodium hypochlorite solution and lastly with double distilled water to remove microscopic entities and other dust particles and later the rhizomes were shade dried for 45 days at room temperature (28 ± 5°C) Turner et al. (2003). The dried materials were then crushed using a blender and made into fine powder.

### 2.2 Aqueous Rhizome Extract Preparation

The powdered sample was extracted using the Soxhlet apparatus. Around 60 g of sample was added into thimble for extraction using water as the solvent for 8 h (24 cycles). The obtained extracts were air-dried and stored at 4°C. Further, it was subjected to qualitative and quantitative phytochemical analysis to quantify the presence of various phytochemicals present in the rhizome extract Mudher and Lovestone (2002). Further, the prepared extract was sent for GC-MS (gas chromatography-mass spectrometry) analysis to identify the important phytochemical constituents and functional groups Goedert et al. (1991).

### 2.3 Preparation of Hydroyapatite Nanoparticles (AC-HAp NPs)

1 M CaCl_2_ and 0.6 M Na_2_HPO_4_ were prepared using leaf extract as the solvent and separately raised to pH 10.0 using 0.8 M NaOH solution for the synthesis of HAp nanospheres. The CaCl_2_ solution was then aggressively agitated at room temperature with a magnetic stirrer, and then Na_2_HPO_4_ solution was added drop by drop to generate a gelatinous precipitate. The formation of precipitation of HAp is described as follows:

10 CaCl_2_ + 6 Na_2_HPO_4_ + 8 NaOH → Ca_10_(PO_4_)_6_(OH)_2_ + 20 NaCl + 6 H_2_O

The precipitate formed was centrifuged to eliminate byproducts before being dried in a hot air oven at 130°C for 6 h and resulting in a dry cake which was crushed to form powder Iqbal et al. (2005). In addition, for comparison, HAp without rhizome extract was made and termed control (pure HAp).

### 2.4 Anti-Acetylcholinesterase Inhibition Assay

Ellman’s method was slightly modified to measure AChE inhibition. Briefly, 150 μl of 0.1 M sodium phosphate buffer (pH 8.0), 10 μl of test chemical solution, and 20 μl of AChE enzyme solution (0.1 units/mL) were combined and incubated at 25°C for 15 min. After that, 10 μl of DTNB (10 mM) (5,5-dithio-bis-(2-nitrobenzoic acid)) was added, and the reaction was started by adding substrate (10 μl of ATCI (acetylthiocholine iodide), 14 mM solution). The formation of the colored product, 5-thio-2-nitrobenzoate anion generated by the reaction of DTNB and thiocholine, which is released by the ATCI’s hydrolysis, can be used to determine the enzyme’s hydrolysis. After 10 min, the colored product was detected at 410 nm wavelength. Tacrine was utilized as a positive control Kametani and Hasegawa (2018). Inhibition (%) was estimated using the following equation:

$$InhibitionActivity\left(\%\right)=\left[1-\frac{Absorbanceofsample}{Absorbanceofcontrol}\right]\times 100$$

### 2.5 In Silico Bioinformatics Studies

#### 2.5.1 Lead Optimization

GC-MS analysis is one of the fast, best and accurate technique used to detect various compounds that includes organic acids, long chain hydrocarbons, alcohols, steroids, amino acids, nitro compounds, alkaloids and esters Pradeep et al. (2020). The GC-MS analysis of aqueous extract of rhizomes of the *Acorus calamus* detected the presence of 110 compounds among which a library of 20 molecules were created based on the review of literature Singh et al. (2010). Subsequently, all 20 molecules were analyzed for their bioactivity through *in silico* molecular docking studies.

The 2D chemical structure of all the 20 molecules were sketched using ChemSketch software Tian et al. (2010). These files were further converted to 3D structures (pdb format) using OpenBabel GUI2.4.1 software Obulesu and Rao (2011). Before carrying out the molecular docking studies, the geometry of all the structures was cleaned using ArgusLab program Jaiswal et al. (2011).

#### 2.5.2 Protein Preparation

Proteins/enzymes that are mainly involved in amyloid hypothesis of AD are BNDF (brain-derived neurotophic factor), APOE4, PKC-γ (protein kinase c), BACE1 and γ-secretase. By altering synaptic plasticity, BDNF plays a fundamental role in cognition, learning, and memory formation, making it a critical molecule in dementia and neurodegenerative illnesses Prasad et al. (2021). The biggest genetic risk factor for Alzheimer’s disease is APOE4. It is important for the metabolism of lipids such as cholesterol and for the repair of neuronal injury in the brain Kollur et al. (2021). PKC isoforms have crucial functions as tau kinases in addition to their role in memory formation. PKC-γ is involved in the maintenance of synaptic plasticity Prasad et al. (2020c). BACE1 (β-secretase 1) catalyses the amyloid precursor protein’s initial cleavage to produce Aβ proteins. As a result, inhibiting BACE1 activity could prevent one of the earliest pathogenic events in Alzheimer’s disease Ankegowda et al. (2020). γ-Secretase is a protease complex that cuts the transmembrane domain of the APP to create the amyloid β-protein (Aβ), an aggregation-prone product that builds up in the brain of Alzheimer’s patients Desikan et al. (2009). The above mentioned protein play very important role in memory and cognition functions thus, all the 5 enzymes were selected for the *in silico* inhibition studies to dock the screened phytocompounds against them.

The three dimensional structures of BNDF, APOE4, PKC-γ, BACE1 and γ-secretase with their respective PDB IDs such as 1B8M, 1GS9, 3PFQ, 4L7G and 5A63, required for the *in silico* studies were obtained from Protein Data Bank (PDB) [https://www.rcsb.org/], a protein structural database Tiraboschi et al. (2004). Before beginning with the docking analysis, all of the protein structures were refined and energy-optimized Mendez (2006). The cleanup of the proteins was accomplished by finishing incomplete residues with hydrogen atoms. External ligands and non-essential ions were removed from the protein structure Waldemar et al. (2007).

#### 2.5.3 Protein Structure Validation

Using the PROCHECK module of the PDBSum server [https://servicesn.mbi.ucla.edu/PROCHECK/], the stereochemical stability of the predicted models was further verified using various protein quality-based parameters such as percentage of residues lying in favored and allowed regions, number of glycine and proline residues, and orientation of dihedral angles including phi (ϕ) and psi (ψ), as well as backbone conformation Schroeter et al. (2009).

#### 2.5.4 Binding Site Prediction

Residues in the protein interacting with the ligand is termed as a binding site of that protein. This binding site was predicted using the CASTp 3.0 server (http://sts.bioe.uic.edu/castp/index.html?4jii) which stands for Computed Atlas of Surface Topography of proteins. Surface pockets and interior cavities are identified and measured by CASTp Jain et al. (2021). The modeled protein is used to predict ligand binding sites, and the server identifies the amino acids that are relevant for binding interactions.

#### 2.5.5 Molecular Docking Studies

**FIGURE 1:**
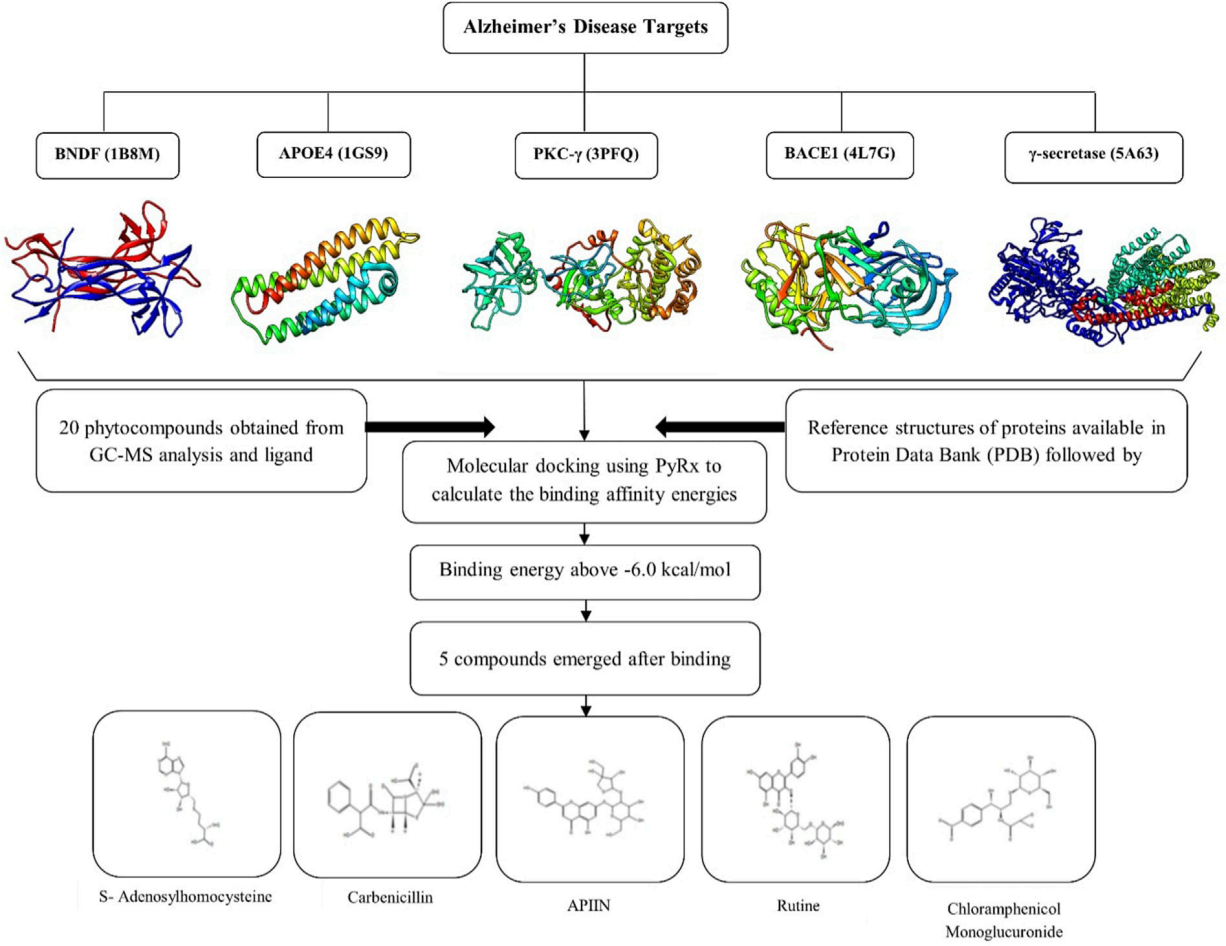
Graphical representation of i*n silico* analysis carried out in the present study and the 3D structure of selected proteins.

MD is a technique for studying the molecular behavior of target proteins when they bind. It is a tool widely utilized in drug development. PyRx 0.8 [https://pyrx.sourceforge.io/], a virtual screening tool was used to accomplish molecular docking Prasad et al. (2020a). A genetic algorithm is an effective approach for searching the docked conformer’s space globally. It also allows for the existence of a population of solutions, which can evolve through processes like ‘breeding’ and ‘mutation’ Prasad et al. (2020b). Poor solutions are extinguished, while good ones are passed down to future generations. In a few tens of generations, such algorithms may usually obtain an excellent answer Uppar et al. (2021). The MD results were analyzed for their bonded and non-bonded interactions using Discovery Studio 3.1 (Accelrys, San Diego, United States) visualization software Avinash et al. (2021). The whole process is depicted in Figure 1

### 2.6 Computational Pharmacokinetics Analysis

It is critical to learn about the pharmacokinetics, or the fate of a molecule in the body, during the creation of a novel therapeutic medicine. Individual indices known as Absorption, Distribution, Metabolism, Excretion, and Toxicity (ADMET) factors are typically used to do this. As an alternative to employing experimental approaches to determine these parameters, computer models are commonly used. Chemicalize, a software developed by ChemAxon [http://www.chemaxon.com], and the internet available SwissADME program were used to estimate some ADME parameters in this study Daina et al. (2017). Additional information about the Pharmacokinetics parameters and the ADMET properties were obtained by resorting to pkCSM Pires et al. (2015), a software for the prediction of small-molecule pharmacokinetic properties using SMILES [https://biosig.unimelb.edu.au/pkcsm/] (accessed, June 2021). Molinspiration Cheminoformatics’ freely available Molinspiration software [https://www.molinspiration.com/] (accessed, June 2021) was used to conduct similarity searches in the chemical space of compounds with molecular structures comparable to those being researched and to predict bioactivity ratings for a variety of pharmacological targets. A Webtool named SwissTargetPrediction for efficient prediction of protein targets of small molecules was used for the determination of the potential bioactivity of the five ligands considered in this study Daina et al. (2019). The associated website allows the estimation of the most probable macromolecular targets of a small molecule, assumed as bioactive.

### 2.7 Conceptual DFT Studies

The molecular energy, electronic density, and orbital energies of a particular system, including the Highest Occupied Molecular Orbital (HOMO) and the Lowest Unoccupied Molecular Orbital (LUMO) were determined using the Kohn-Sham (KS) approach Lewars (2003); Young (2001); Jensen (2007); Cramer (2004) while making use of the Conceptual DFT (CDFT) methodology Parr and Yang (1989); Chermette (1999); Geerlings et al. (2003, 2020); Toro-Labbé (2007); Chattaraj (2009); Chakraborty and Chattaraj (2021). The conformers of the compounds studied in this work were determined using MarvinView 17.15 from ChemAxon [http://www.chemaxon.com] by using the entire MMFF94 force field to perform Molecular Mechanics calculations Halgren (1996a,b, 1999); Halgren and Nachbar (1996); Halgren (1996c). This was followed by a geometry optimization and frequency calculation by means of the Density Functional Tight Binding (DFTBA) methodology Frisch et al. (2016). This last step was required for the verification of the absence of imaginary frequencies as a check for the stability of the optimized structures as being a minimum in the energy landscape. The electronic properties and the chemical reactivity descriptors of the studied molecules involved the use of MN12SX/Def2TZVP/H2O model chemistry Peverati and Truhlar (2012); Weigend and Ahlrichs (2005); Weigend (2006) on the optimized molecular structures due to is ability in the verification of the ‘Koopmans in DFT’ (KID) protocol Flores-Holguín et al. (2019b); Flores-Holguín et al. (2019d); Frau and Glossman-Mitnik (2018a); Frau and Glossman-Mitnik (2018b); Frau and Glossman-Mitnik (2018c); Frau and Glossman-Mitnik (2018d); Frau and Glossman-Mitnik (2018e); Frau and Glossman-Mitnik (2018f); Flores-Holguín et al. (2019a); Frau et al. (2019); Flores-Holguín et al. (2019c), Flores-Holguín et al. (2020c); Flores-Holguín et al. (2020a); Flores-Holguín et al. (2020b); Flores-Holguín et al. (2021) using Gaussian 16 Frisch et al. (2016) and the SMD model for the simulation of the solvent Marenich et al. (2009). This model chemistry considers the MN12SX screened-exchange density functional Peverati and Truhlar (2012) together with the Def2TZVP basis set Weigend and Ahlrichs (2005); Weigend (2006) and in all cases the charge of the molecules is equal to zero while the radical anion and cation have been considered in the doublet spin state.

## 3 Results

### 3.1 SEM Analysis

**FIGURE 2:**
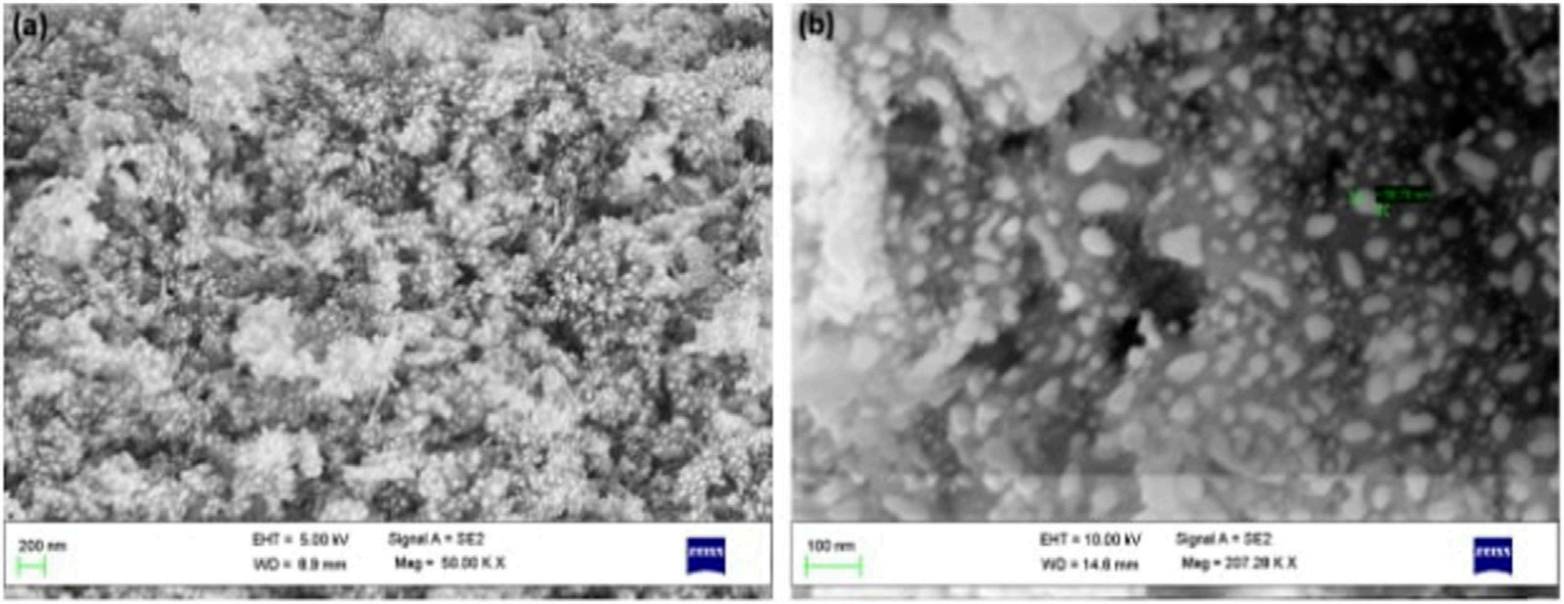
SEM images of *A. calamus* rhizome extract derived HAp NPs.

**FIGURE 3:**
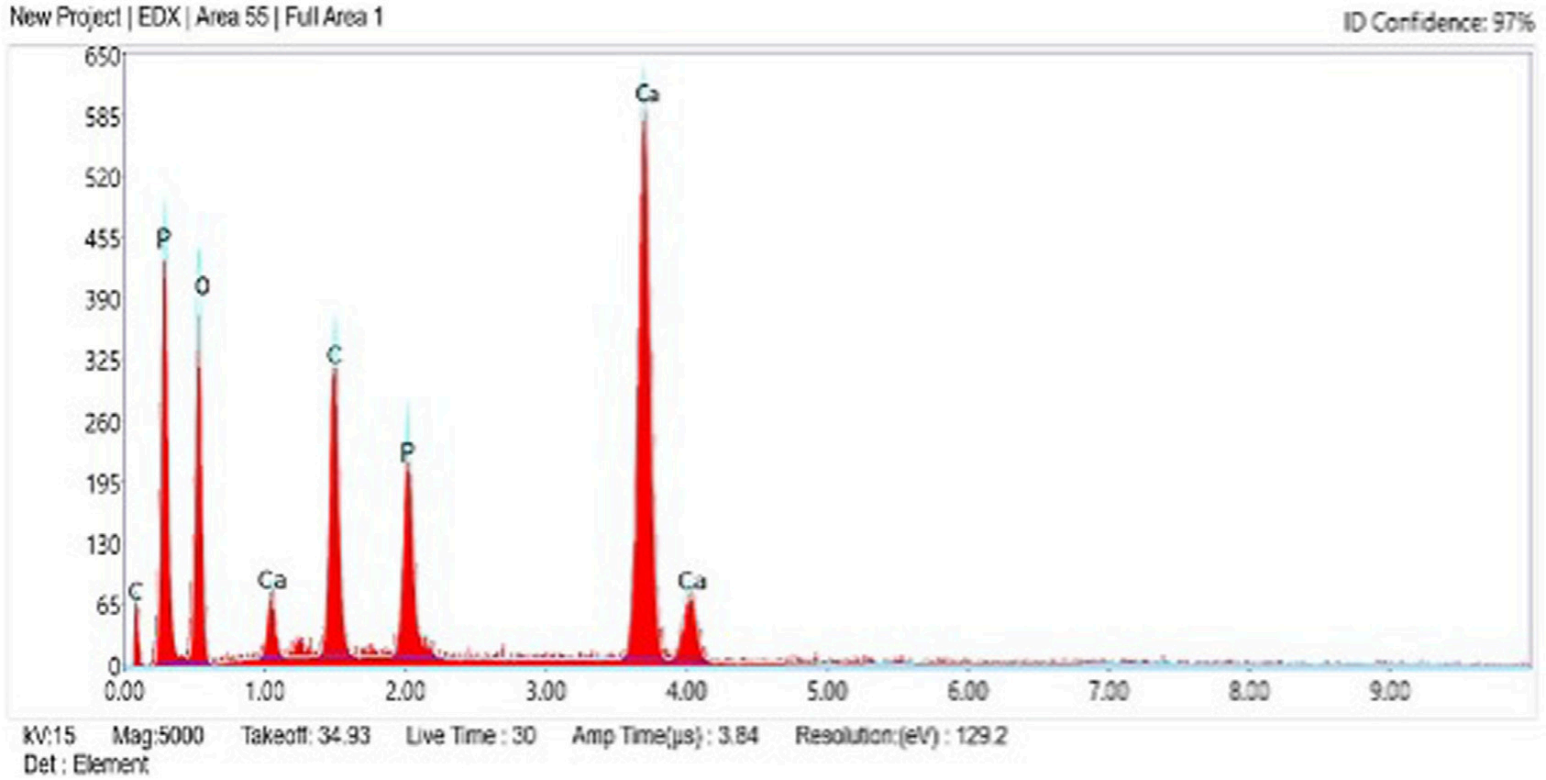
EDX spectra showing the elements present in as-prepared HAp NPs.

The surface morphology of as-prepared AC-HAp NPs showed spherical shaped particles which are highly agglomerated. The average particles size ranged between 30 and 50 nm (Figure 2). Further, EDAX analysis was carried out to explore the composition of the AC-HAp NPs. Figure 3 depicts the EDX spectra of as-obtained AC-HAp NPs showing the characteristic peaks of Ca, P and O with the atomic and weight percentages of the elemental particles providing the mean relative calcium to phosphate ratios, and was found to be 1.68, which is quite close to the Ca/P ratio of the human bone.

### 3.2 X-ray Diffraction Analysis

**FIGURE 4:**
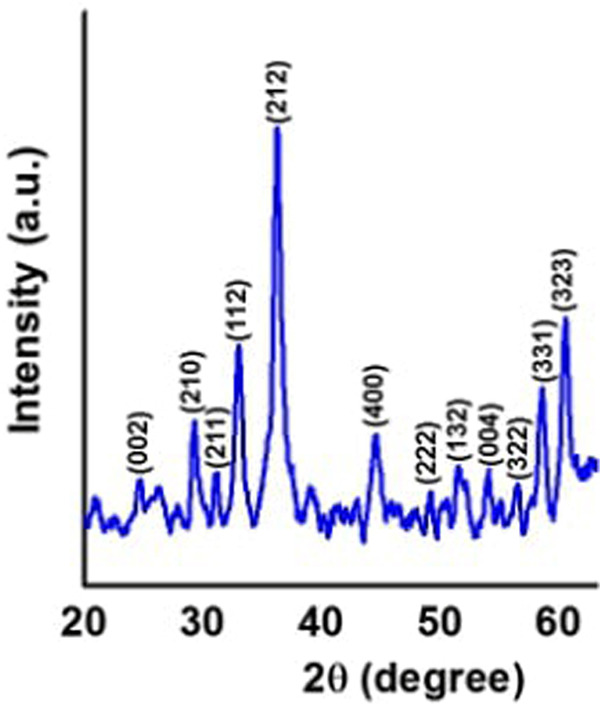
XRD diffraction pattern of as-prepared AC-HAp NPs.

The crystalline phases of the as-prepared AC-HAp NPs was determined using XRD diffraction pattern (Figure 4). The position of observed diffraction peaks are in good agreement with the JCPDS (89-6438). The peaks observed at 2θ = 27.8°, 30.1°, 33.3°, 35.1°, 36.2°, 45.8°, 49.8°, and 60.2° corresponds to the (hkl): (002), (210), (211), (112), (212), (400), (222), and (323), matching exactly with the hexagonal system with primitive lattice. Furthermore, the average particle size of the as-prepared AC-HAp NPs was said to 36 mm, which was calculated (using FWHM) by Scherrer’ formulam D = k λ/β cosθ Ankegowda et al. (2020).

### 3.3 TEM Analysis

**FIGURE 5:**
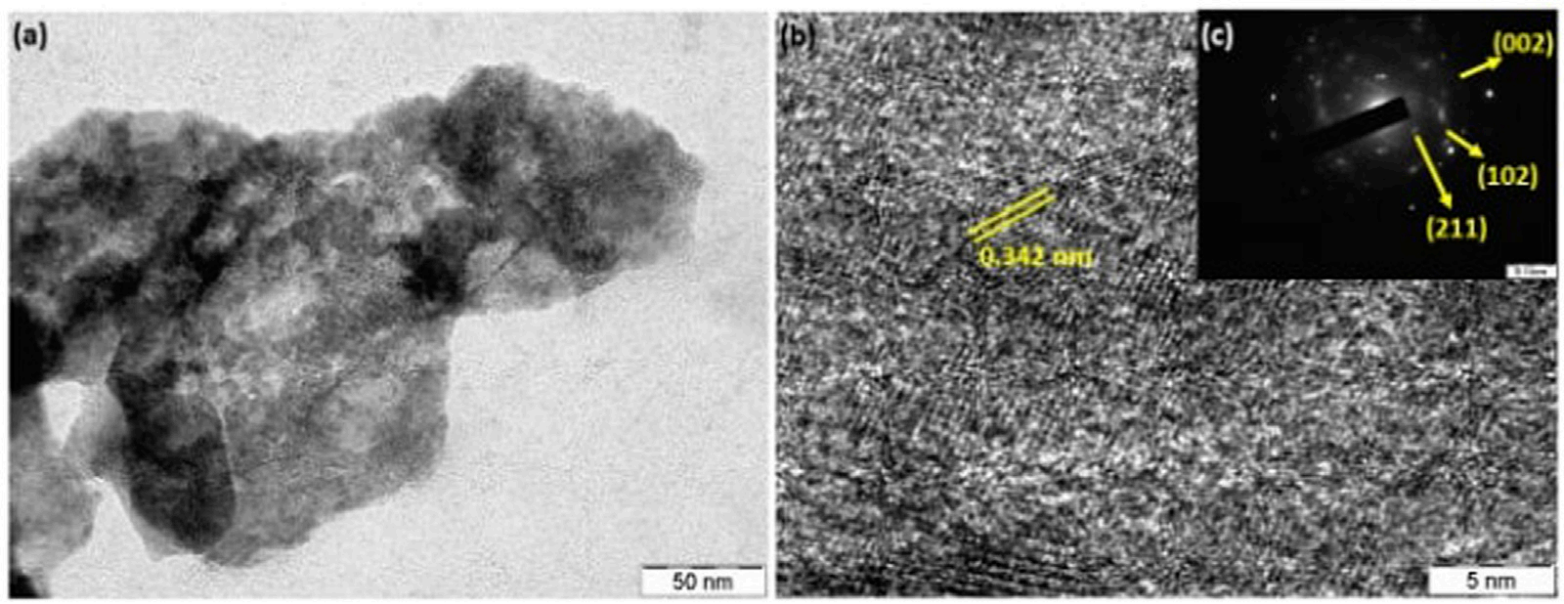
**(A)** TEM; **(B)** HR-TEM, and **(C)** SAED pattern of as-prepared AC-HAp NPs.

The structure and morphology of as-prepared AC-HAp NPs was determined by Transmission electron microscopy (TEM). The TEM images as shown in Figure 5, reveals that the average sizes were between 30 and 40 nm. The spherical nature of AC-HAp NPs is evident from the TEM image. Moreover, the aggregate blocks with porous structure of the material can be seen form TEM image. Further, the HR-TEM image showed that the inter-planer spacing between two lattice fringes was 0.342 nm (Figure 5B), corresponding to (102) lattice plane of AC-HAp NPs, and same has been confirmed by SAED pattern, which shows the crystalline structure of as-prepared AC-HAp NPs (Figure 5C).

### 3.4 AChE Inhibition

By inhibiting AChE of the cholinergic synapse, AChE inhibitors enhance acetylcholine levels by inhibiting AChE of the cholinergic synapse thus enhancing the function and relieving the symptoms of neurological illnesses, including Alzheimer’s disease. In addition to alkaloid-derived chemicals as the most well-known natural AChE inhibitors, plant-derived extract are also a major source of AChE inhibitors. HAp NPs from the rhizome of *A. calamus* has been demonstrated to inhibit AChE. As a result, AC-HAp NPs of the *A. calamus* rhizome were found to have AChE inhibitory action.

**FIGURE 6:**
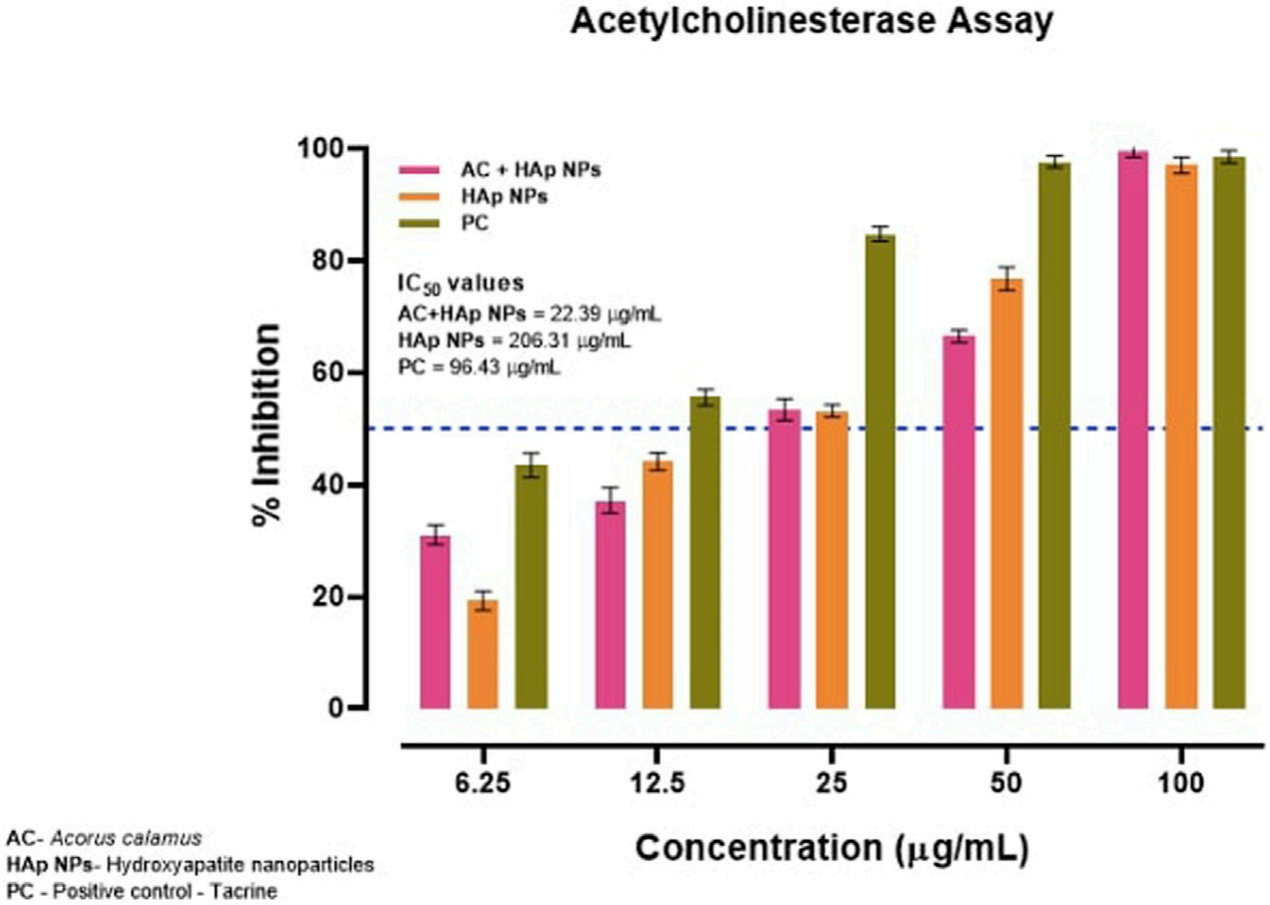
Anti-acetylcholinesterase activity of HAp NPs derived from *A. calalmus*, pure HAp NPs and Tacrine as positive control.

Surprisingly, the anti-AChE activity was evidently greater with AC-HAp NPs when compared to positive control Tacrine and pure HAp NPs with IC50 value of about 22.39 μg/ml (Figure 6).

### 3.5 MD Interactions

**FIGURE 7:**
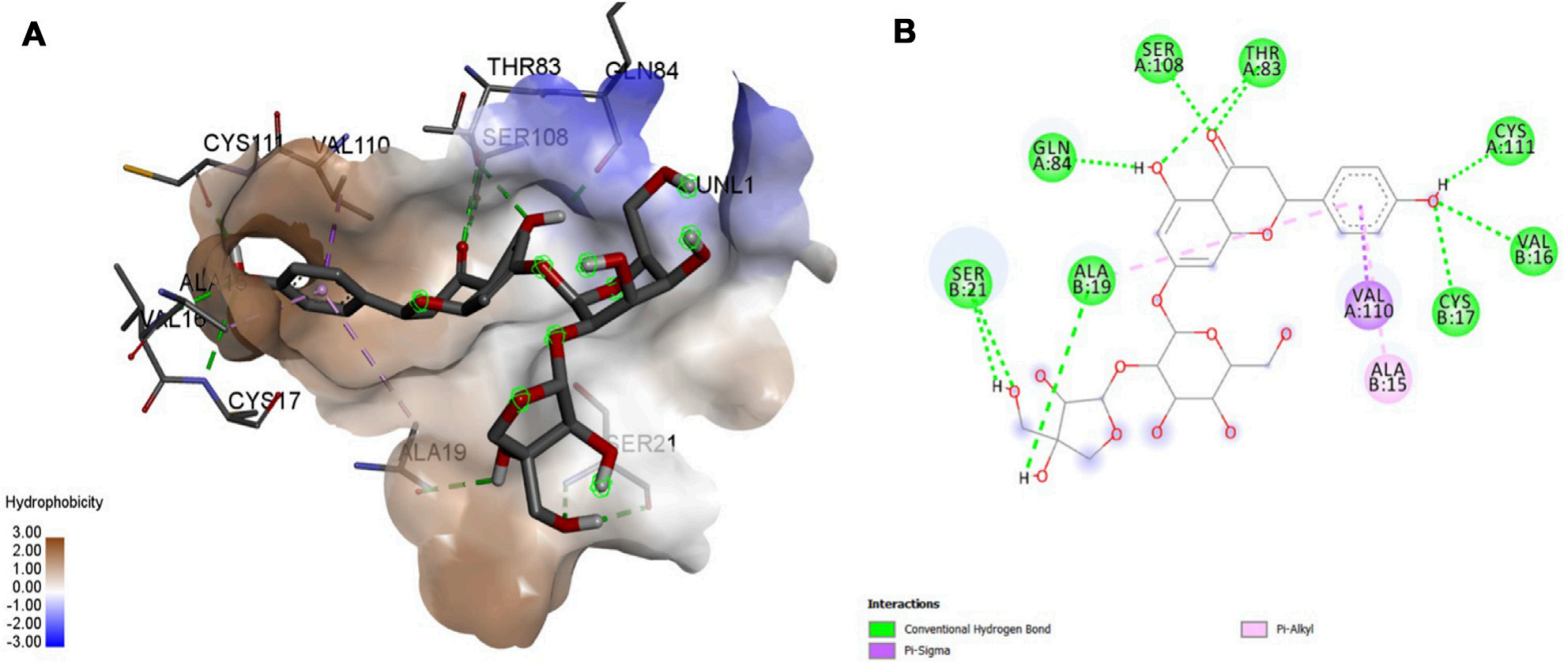
Molecular docking interaction analysis of protein 1B8M, **(A)**: 3D interactions and **(B)**: 2D interactions have been represented between 1B8M-Apiin complex structures.

**FIGURE 8:**
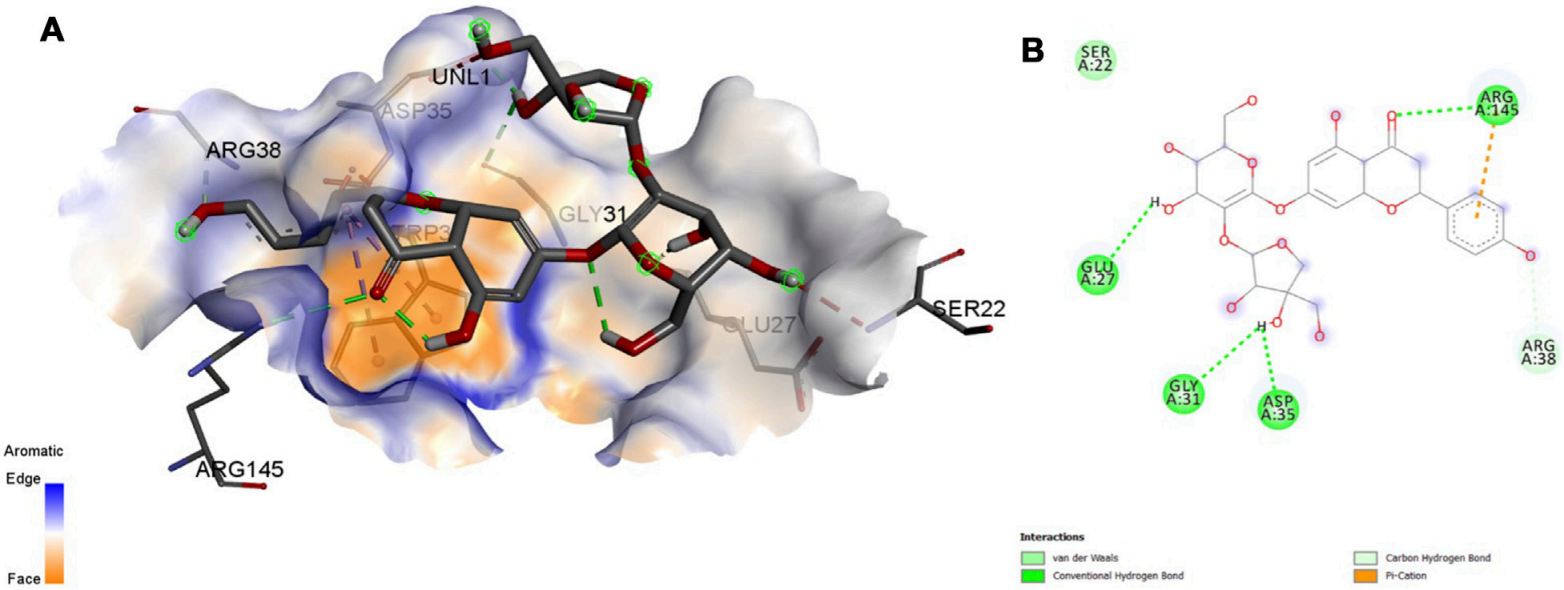
Molecular docking interaction analysis of protein 1GS9, **(A)**: 3D interactions and **(B)**: 2D interactions have been represented between 1GS9-Apiin complex structures.

**FIGURE 9:**
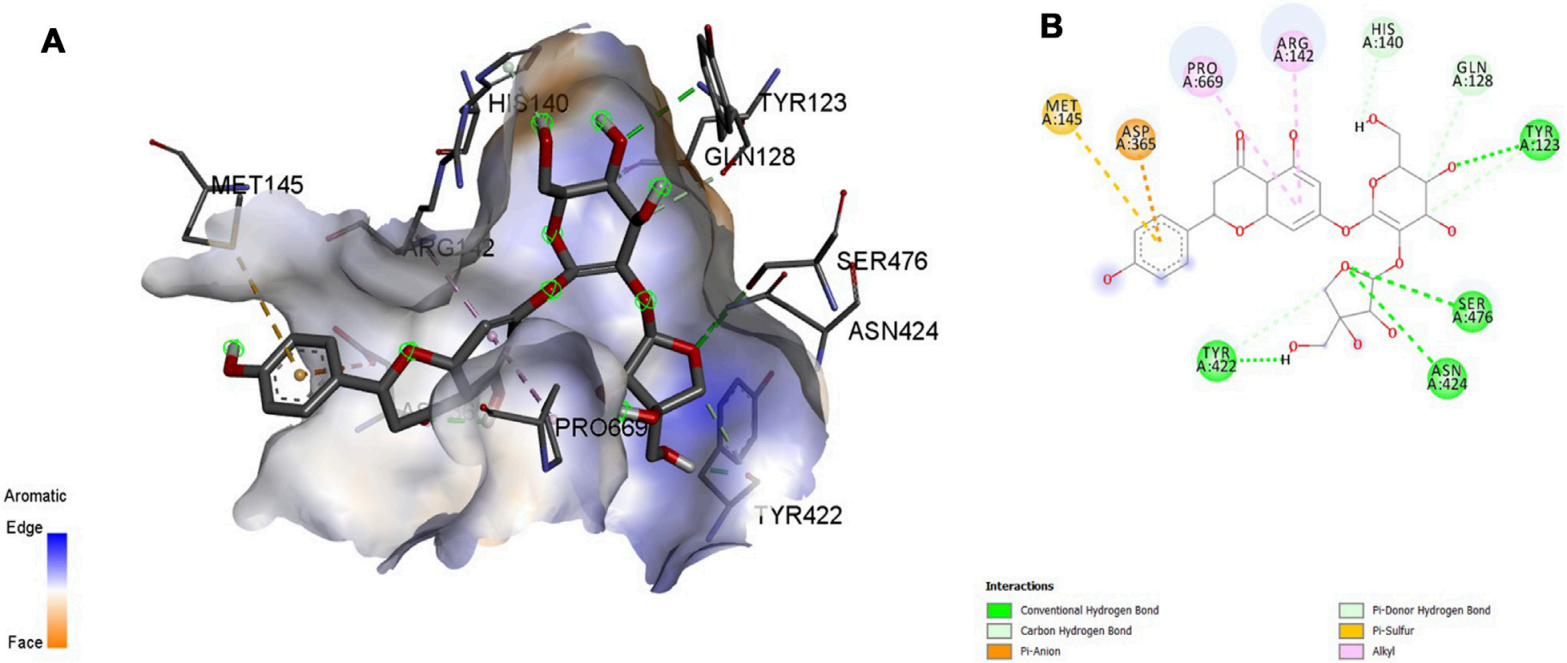
Molecular docking interaction analysis of protein 3PFQ, **(A)**: 3D interactions and **(B)**: 2D interactions have been represented between 3PFQ-Apiin complex structures.

**FIGURE 10:**
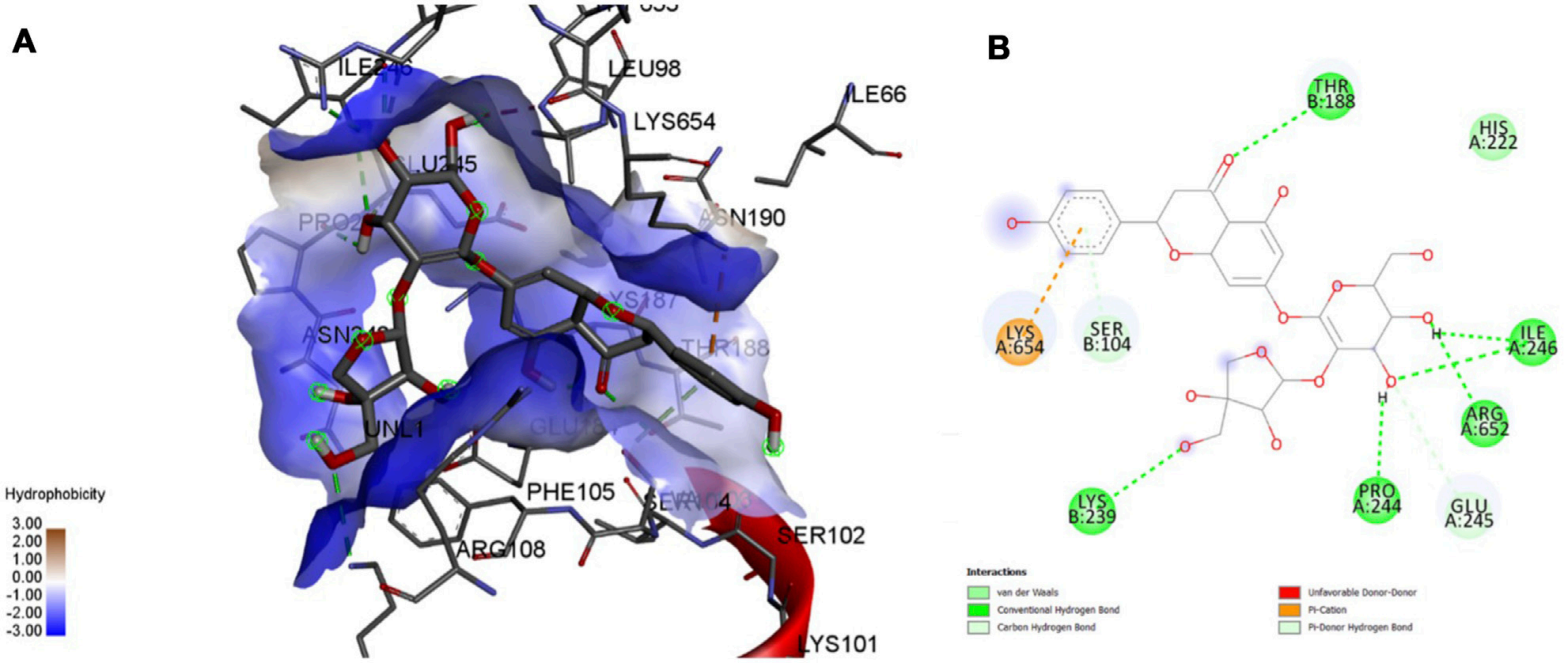
Molecular docking interaction analysis of protein 5A63, **(A)**: 3D interactions and **(B)**: 2D interactions have been represented between 5A63-Apiin complex structures.

**FIGURE 11:**
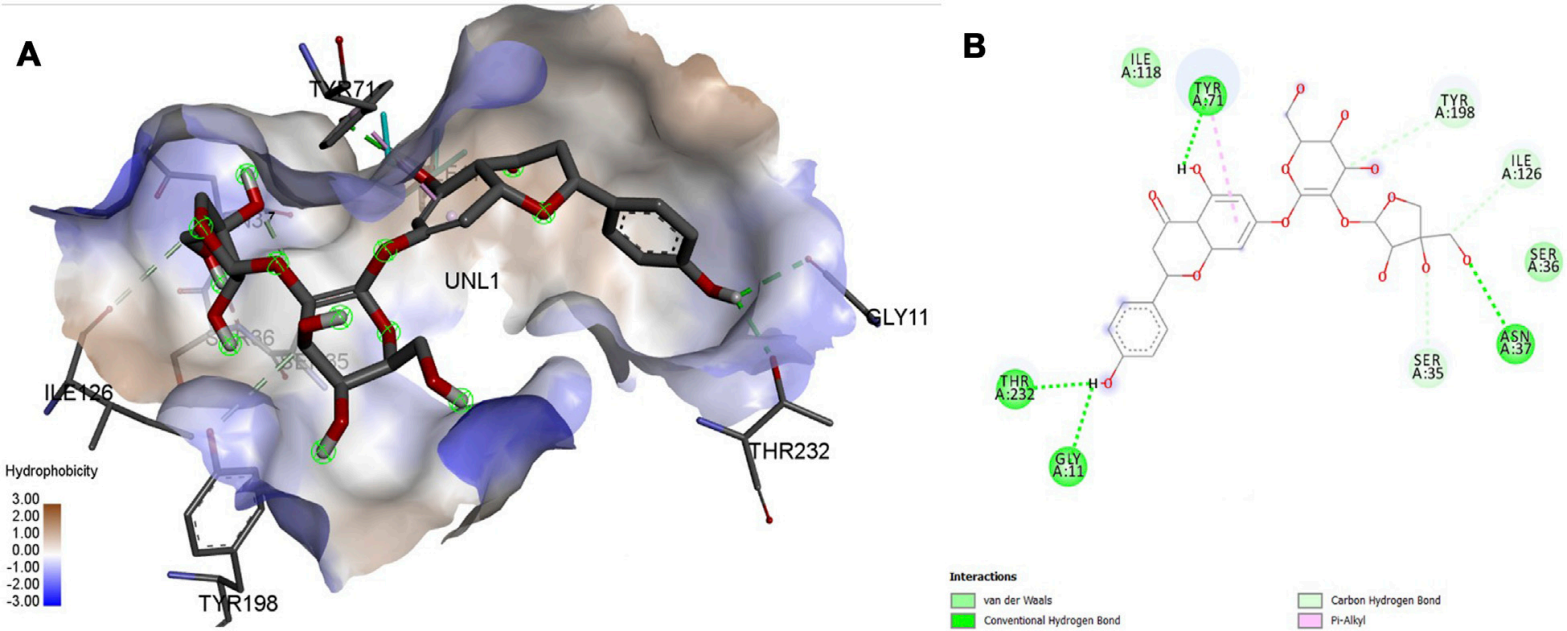
Molecular docking interaction analysis of protein 4L7G, **(A)**: 3D interactions and **(B)**: 2D interactions have been represented between 4L7G-Apiin complex structures.

Interaction affinity describes the strength of protein-ligand binding. The binding affinity is determined by the strength of the attractive force between the protein and the ligand. The best molecularly docked poses were analyzed and visualized. The docking procedure was validated before the ligands were screened. The optimum ligand-protein complex orientations were investigated. The docking score was used to identify the excellent docking conformation. The binding affinity of a specific protein-ligand complex with a known 3D structure is computed using the binding energy score. Van der Waals interactions, hydrogen bonding and hydrophobic effects are all included in the binding energy (Table 1). The 3D and 2D interactions between all the protein-Apiin complexes were analyzed and its images were taken using Discovery Studio 3.1 visualization software from Figures 7–11.

**TABLE 1:** Protein-ligand complex binding energy and interaction details.

Protein ID	Ligand name	Binding affinity (Kcal/mol)	Number of hydrogen bonds	Residues forming hydrogen bonds
1B8M	S-Adenosylhomocysteine	−6.0	7	THR-83, GLN-84, SER-21, ALA-19, CYS-119
	Carbenicillin	−7.4	8	THR-59, THR-83, GLN-84, TYR-86, SER-108, THR-117
	Apiin	−7.2	10	SER-21, ALA-19, GLN-84, SER-108, THR-83, CYS-111
				VAL-16, CYS-17
	Rutine	−6.9	11	SER-17, CYS-13, TYR, 54, THR-56, GLN-94, LYS-93
				CYS-13, ASP-14
	Chloramphenicol Monoglucoronide	−7.0	10	GLU-9, SER-11, ASP-14, SER-15, LYS-93, TYR-96
1GS9	S-Adenosylhomocysteine	−5.7	6	GLY-23, GLU-27, ASP-36, ASP-153, GLN-156
	Carbenicillin	−6.8	3	Trp-34, ASP-35, GLN-156
	Apiin	−6.0	6	GLU-27, ARG-145, GLN-156
	Rutine	−6.6	4	VAL-103, SER-104, ARG-108, THR-188, HIS-222
				GLU-245, ILE-246
	Chloramphenicol Monoglucoronide	−6.9	3	GLU-27, ARG-145, GLN-156
3PFQ	S-Adenosylhomocysteine	−6.4	9	VAL-103, SER-104, ARG-108, THR-188, HIS-222
				GLU-245, ILE-246
	Carbenicillin	−8.6	7	VAL-103, SER-104, ARG-108, PRO-244, GLU-245
				ARG-652
	Apiin	−9.0	9	SER-104, THR-188, LYS-239, PRO-244, GLU-245
				ILE-246, ARG-652
	Rutine	−9.5	8	SER-67, SER-102, VAL-103, ARG-108, THR-188, LYS-654
				LYS-654, ARG-657, ARG-659
	Chloramphenicol Monoglucoronide	−8.6	9	VAL-103, SER-104, ARG-108, GLU-184, HIS-222, ILE-246
				ARG-652
5A63	S-Adenosylhomocysteine	−7.7	7	LEU-348, TYR-422, VAL-423, GLY-426, ASP-427, ASP-470
	Carbenicillin	−7.2	2	TYR-422, MET-473
	Apiin	−9.0	8	TYR-123, GLN-128, HIS-140, TYR-422, ASN-424, SER-476
	Rutine	−7.8	8	LEU-348, GLY-351, GLY-354, GLY-426, ASP-427, ASP-470
	Chloramphenicol Monoglucoronide	−7.4	5	TYR-123, GLN-128, GLY-129, LYS-141, SER-476
4L7G	S-Adenosylhomocysteine	−7.8	5	SER-35, ASN-37, TYR-71, ILE-126
	Carbenicillin	−7.4	5	GLY-11, TYR-71, THR-232
	Apiin	−7.4	4	GLY-11, SER-35, SER-36, ASN-37, TYR-71, ILE-126
				TYR-198, THR-232
	Rutine	−9.2	8	ASP-32, SER-35, ASN-37, ALA-39, TYR-198, LYS-224
				THR-231, ARG-235
	Chloramphenicol Monoglucoronide	−10.7	10	GLY-11, SER-35, TYR-71, THR-72, GLN-73, GLY-230
				THR-232

The MD studies revealed that only 5 molecules out of 20 molecules has the ability to bind to the active site with the selected targets by forming greater binding affinity and least binding energy against the targets. Also, all the 25 protein-ligand complexes were capable of forming a very good amount of bonded and non-bonded interaction between them. Therefore, all the five phytoconstituents obtained from the GC-MS analysis of aqueous extracts of A. calamus were able to form a great interaction with all the 5 selected targets and thus showed the *in silico* inhibition activity against AD.

### 3.6 Computational Pharmacokinetics Report

The Bioactivity Scores, that is a measure of the ability of the molecules to behave or interact with different receptors, for the five ligands are presented in Table 2

**TABLE 2:** Bioactivity scores of the studied molecules calculated on the basis of the GPCR ligand, ion channel modulator, nuclear receptor ligand, kinase inhibitor, protease inhibitor, and enzyme inhibitor interactions.

Property	S-adenosyl homocysteine	Carbenicillin	Apiin	Rutine	Chloramphenicol monoglucoronide
GPCR Ligand	1.04	0.05	0.18	−0.05	0.08
Ion Channel Modulator	0.44	−0.40	−0.17	−0.52	−0.06
Nuclear Receptor Ligand	0.47	−0.75	0.09	−0.14	−0.28
Kinase Inhibitor	−1.18	−0.37	0.18	−0.23	−0.12
Protease Inhibitor	0.51	0.85	0.17	−0.07	0.08
Enzyme Inhibitor	1.23	0.30	0.42	0.12	0.28

A chemical with a bioactivity score more than 0 is predicted to have significant biological activities, while values between −0.50 and 0.00 are moderately active. The molecular system is considered inactive if the bioactivity score is less than −0.50. The findings clearly show that the drug complexes’ physiological activities may be mediated by many pathways, including interactions with GPCR ligands, protease inhibitors, and other enzymes. The results from Table 2 indicate that S-Adenosylhomocysteine will mostly act as a GPCR ligand, an enzyme inhibitor and a protease inhibitor. For the case of Carbenicillin, the main interaction are going to be as a protease and enzyme inhibitor. Lastly, by considering the Apiin, Rutine and Chloramphenicol monoglucoronide ligands, they may be regarded as enzyme inhibitors, and with the exception of Rutine, also as protease inhibitors.

An ADMET study is the assessment of pharmacokinetics of a drug which stands for Absorption, Distribution, Metabolism, Excretion and Toxicity. The prediction of the fate of a drug and the effects caused by a drug inside the body, such as how much drug is absorbed if administered orally and how much is absorbed in the gastrointestinal tract, is an indispensable part of drug discovery. In a similar way, if the absorption is poor, its distribution and metabolism would be affected, which can lead to causing neurotoxicity and nephrotoxicity.

The computed ADMET properties of the five studied ligands are presented in Table 3.

**TABLE 3:** ADMET properties of the five studied ligands.

Property	S-adenosyl homocystein	Carbenicillin	Apiin	Rutine	Chloramphenicol monoglucoronide
	Absorption				
Caco-2 Permeability	−0.506	0.377	0.737	−0.763	−0.868
Intestinal Absorption	27.464	23.953	29.350	28.135	0.000
Skin Permeability	−2.735	−2.735	−2.735	−2.735	−2.735
P-glycoprotein Substrate	Yes	Yes	Yes	Yes	Yes
P-glycoprotein I Inhibitor	Yes	No	No	No	No
P-glycoprotein II Inhibitor	No	No	No	No	No
	Distribution				
VDss	−0.575	−1.804	−0.108	0.013	−2.144
Fraction Unbound	0.559	0.427	0.175	0.292	0.427
BBB Permeability	−1.630	−1.051	−1.523	−2.080	−5.744
CNS Permeability	−4.090	−3.572	−5.144	−5.744	−4.556
	Metabolism				
CYP2D6 Substrate	No	No	No	No	No
CYP3A4 Substrate	No	No	No	No	No
CYP1A2 Inhibitor	No	No	No	No	No
CYP2C19 Inhibitor	No	No	No	No	No
CYP2C9 Inhibitor	No	No	No	No	No
CYP2D6 Inhibitor	No	No	No	No	No
CYP3A4 Inhibitor	No	No	No	No	No
	Excretion				
Total Clearance	0.721	0.081	0.117	−0.200	0.373
Renal OCT2 Substrate	No	No	No	No	No
	Toxicity				
AMES Toxicity	No	No	No	Yes	No
Maximum Tolerated Dose	0.514	1.717	0.516	0.427	0.759
hERG I Inhibitor	No	No	No	No	No
hERG II Inhibitor	No	No	Yes	Yes	No
Oral Rat Acute Toxicity	2.403	1.856	2.417	2.445	2.439
Oral Rat Chronic Toxicity	2.771	2.956	5.414	5.414	5.158
Hepatotoxicity	Yes	Yes	No	No	No
Skin Sensitisation	No	No	No	No	No
*T. Pyriformis* Toxicity	0.285	0.285	0.285	0.2.85	0.2.85

A chemical can reach a tissue if it is injected into the bloodstream. Before being taken up by target cells, a drug is usually given through mucous surfaces such as the digestive tract, i.e. intestinal absorption. Drug absorption is limited following oral delivery due to poor substance solubility, intestinal transit time, gastric emptying time, difficulty permeating the intestinal wall, and chemical instability in the stomach. Absorption is important because it affects the bioavailability of a chemical. For medications with low absorption, oral delivery, such as inhalation or intravenously, is less desirable Jujjavarapu et al. (2019); Pires et al. (2015). For projected values >0.90, a substance is deemed to have a high Caco-2 permeability across the human intestinal mucosa, being Apiin with a value of 0.737 the only drug that could be considered in this regard. In most cases, the gut is the principal location of medication absorption from an orally delivered solution. Intestinal Absorption forecasts the percentage of a substance that will be absorbed through the human intestine, with less than 30% being considered poorly absorbed. Again, Apiin is the only drug that satisfies this requirement, according to Table 3. The model forecast whether or not a particular substance will be a P-glycoprotein substrate. This is verified for all the molecules considered in this study. Modulation of P-glycoprotein-mediated transport has significant pharmacokinetic implications for P-glycoprotein substrates, which might have therapeutic benefits or create contraindications. As a result, this study indicates that none of the molecules will inhibit P-glycoprotein I and II, with the exception of S-Adenosylhomocysteine which will be an inhibitor of P-glycoprotein I. Furthermore, it may be predicted whether a certain substance will be skin permeable. If a chemical has a log Kp > −2.5, it is regarded to have low skin permeability, meaning that all five drug may be useful in the development of transdermal medication administration Pires et al. (2015). The total dose of a drug requires a certain volume to be uniformly distributed in blood plasma known as VDss. The drug will be more distributed in the tissue rather than in the plasma for higher VDss. From Table 3, low values of VDss are found for the five drugs. The efficacy of a given drug may be affected by the degree to which it binds proteins within the blood. The Fraction Unbound predicts the fraction that will be unbound in plasma resulting in the values shown in Table 3. A drug’s ability to cross into the brain is a significant descriptor because it will be able to contribute to the reduction of toxicities and side effects, and is evaluated through the Blood-Brain Permeability parameter. For a given potential therapeutic drug, a logBBB > −0.3 value is estimated to readily cross the blood-brain barrier while molecules with logBBB > −1 will be badly distributed to the brain. The CNS Permeability is another measurement having low values which indicates that these drugs cannot penetrate the Central Nervous System (CNS) Pires et al. (2015). Cytochrome P450 is an important detoxification enzyme in the body, mostly present in the liver, since it oxidizes xenobiotics to enhance excretion Pires et al. (2015). Table 3 shows that none of the studied molecules will be inhibitors or substrates of any P450 cytochrome isoform. Drug clearance happens as a combination of renal and hepatic clearance, and is associated with bioavailability; consequently, it is important for determining dosing rates. The AMES toxicity test utilizes microbes in oder to ascertain a compound’s mutagenesis potential. A positive test shows that the substance is mutagenic; therefore, it could result in cancer. The predictions are negative for all the molecules with the exception of Rutine. The main causes of acquiring long QT syndrome are the blocking of the potassium channels encoded by hERG (the human Ether-a-go-go-Related Gene), which leads to fatal ventricular arrhythmia. The predictions indicate that none of the molecules will be an hERG inhibitor, but Apiin and Rutine will be hERG II inhibitors. The lethal dosage value (LD50) can be assessed in terms of the ORAT (Oral Rat Acute Toxicity) and the ORCT (Oral Rat Chronic Toxicity) parameters. Drug-induced liver injury is a major safety concern for drug development and a significant cause of drug attrition. Thus, Hepatoxicity is related to the disruption of the normal liver function and the predictions for Apiin, Rutine aand Chloramphenicol Monoglucoronide are negative. Skin Sensitization is predicted negative in all cases. T. Pyriformis is a protozoa bacteria whose toxicity is frequently applied as a toxic endpoint. A forecasted value > −0.5 for a given compound is considered toxic Pires et al. (2015).

### 3.7 Conceptual DFT Studies

The calculated global reactivity descriptors: Electronegativity (*χ*), Hardness (*η*), Electrophilicity (*ω*) (all in eV), Softness (S), Nucleophilicity (N), Electrodonating Power (*ω* ^−^), Electroaccepting Power (*ω* ^+^) and Net Electrophilicity (Δ*ω* ^±^) Parr and Yang (1989); Chermette (1999); Geerlings et al. (2003, 2020); Toro-Labbé (2007); Chattaraj (2009); Chakraborty and Chattaraj (2021), estimated following the methodology presented in the 2.7 subsection together with the in-house developed CDFT software tool are displayed in Table 4

**TABLE 4:** Global Reactivity Descriptors of the Five Studied Ligands: Electronegativity (*χ*), Hardness (*η*), Electrophilicity (*ω*) (all in eV), Softness (S) (in eV^−1^), Nucleophilicity (N), Electrodonating Power (*ω* ^−^), Electroaccepting Power (*ω* ^+^) and Net Electrophilicity (Δ*ω* ^±^) (also in eV).

Molecule	*χ*	*H*	*ω*	S	N	*ω* ^−^	*ω* ^+^	Δ*ω* ^±^
S-Adenosylhomocysteine	3.8897	4.2687	1.7722	0.2343	2.7684	5.7561	1.8664	7.6225
Carbenicillin	3.8718	5.6238	1.3328	0.1778	2.1088	4.9530	1.0812	6.0341
Apiin	4.1168	4.6227	1.8332	0.2163	2.3643	6.0136	1.8968	7.9105
Rutine	4.1346	4.1184	2.0754	0.2428	2.5986	6.4756	2.3410	8.8166
Chloramphenicol Monoglucoronide	5.2081	4.2387	3.1996	0.2359	2.8369	9.2682	4.0601	13.3283

As the global hardness *η* can be regarded as a direct measure of the deformation of the electron density and of the chemical reactivity being related to the HOMO-LUMO gap, it can be seen that Carbenicillin will be the less reactive ligand being the others very similar in their reactivity. The electrodonating ability *ω* ^−^ is more important than its electroaccepting power *ω* ^+^ for all the ligands because of their molecular structures. However, after a comparison of the values of *ω* ^−^ and *ω* ^+^ for each molecule, it can be concluded that the reactivity of the Chloramphenicol monoglucoronide will be very different from the other ligands. The electrophilicity *ω* index encompasses the equilibrium between an electrophile’s tendency to acquire extra electron density and a molecule’s resistance to exchanging electron density with the environment Domingo et al. (2016). By studying the electrophilicities of a series of reagents involved in Diels-Alder reactions Domingo et al. (2002); Domingo and Sáez (2009); Pérez et al. (2003), an electrophilicity *ω* scale for the classification of organic molecules as strong, moderate or marginal electrophiles was proposed being *ω* > 1.5 eV for the first case, 0.8 < *ω* < 1.5 eV for the second case and *ω* < 0.8 eV for the last case Domingo et al. (2002); Domingo and Sáez (2009); Pérez et al. (2003). By inspection of Table 4, it can be said that with the exception of Carbenicillin all the ligands may be regarded as strong electrophiles.

Besides the global reactivity descriptors, their local counterparts have been developed to get an idea of the differences in chemical reactivity between the atoms within the molecule. Among these local reactivity descriptors are the Fukui functions Parr and Yang (1989); Chermette (1999); Geerlings et al. (2003) and the Dual Descriptor Toro-Labbé (2007); Morell et al. (2005, 2006); Martínez-Araya (2012a); Martínez-Araya (2012b); Martínez-Araya (2015), which have been defined as: Nucleophilic Fukui Function (NFF) = *f* ^+^(**r**) = *ρ*_*N*+1_(**r**) − *ρ*_*N*_(**r**), Electrophilic Fukui Function (EFF) = *f* ^−^(**r**) = *ρ*_*N*_(**r**) − *ρ*_*N*−1_(**r**), and Dual Descriptor (DD) = Δ*f*(**r**) = ${\left(\partial \;f\left(r\right)/\partial \;N\right)}_{\upsilon \left(r\right)}$ , relating the electronic densities of the neutral, positive and negative species.

**FIGURE 12:**
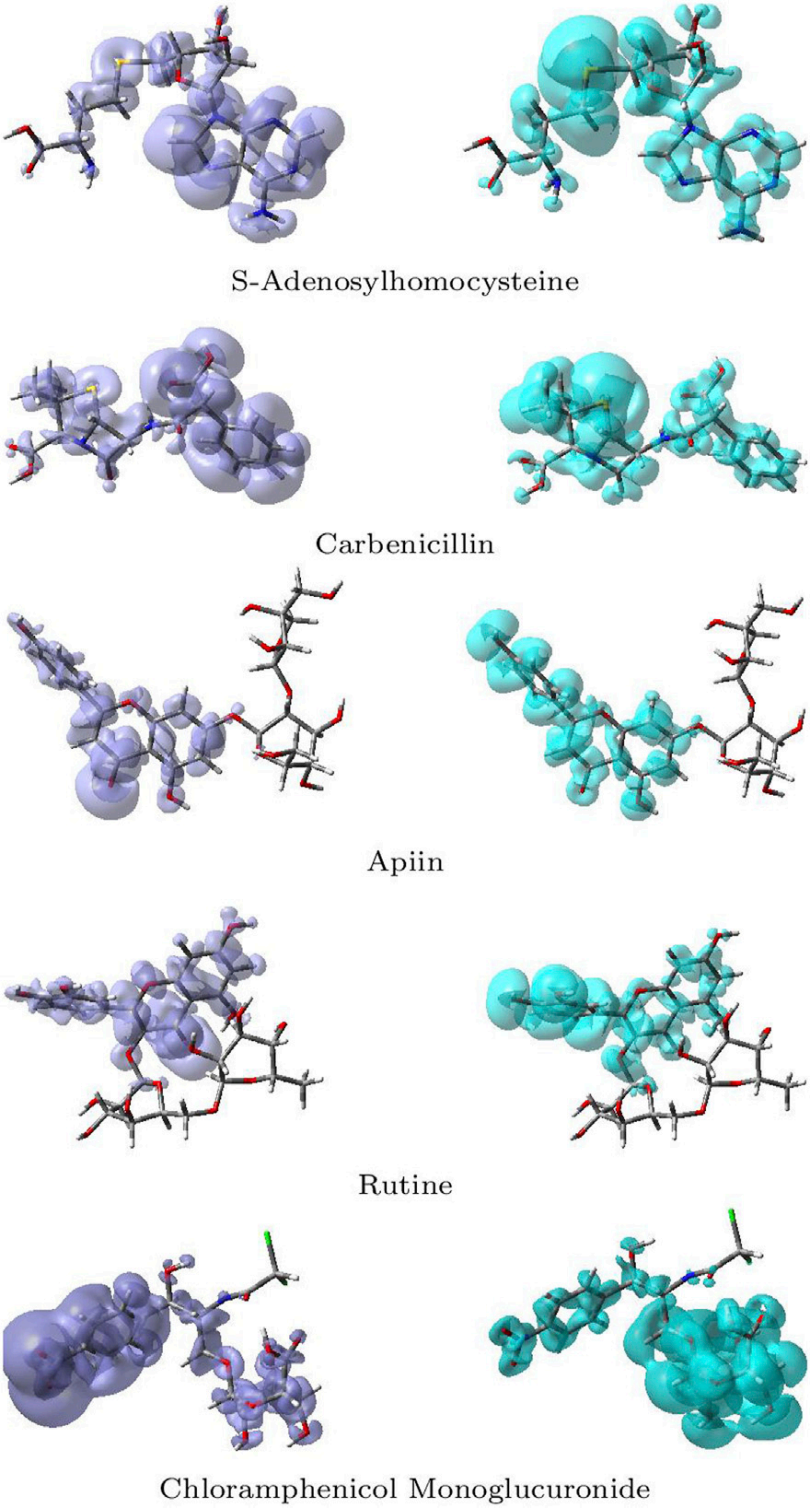
Graphical representations of the dual descriptor DD of the five studied ligands. Left: DD < 0, Right: DD < 0.

The NFF, *f* ^+^(**r**), is associated with the sites within a molecular system which are prone to nucleophilic attacks while the EFF, *f* ^−^(**r**), describes those sites that are more susceptible to electrophilic attacks. Although the NFF and the EFF have been used successfully for the identification of reactive sites, the Dual Descriptor Δ*f*(**r**) or DD, has been shown to describe unambiguously nucleophilic and electrophilic sites within a molecule Martínez-Araya (2015). Graphical representations of the DD for the five studied ligands is displayed in Figure 12 showing the zones where DD > 0 and DD < 0:

Although there is some overlap between the different regions within the ligands, these graphical representations allow to clearly distinguish the regions within the molecules where the Dual Descriptor will be greater or smaller than zero, implying the differences in their chemical reactivities.

## 4 Discussion

Alzheimer’s disease (AD) is a chronic neurodegenerative disorder characterized by the progressive impairment of memory, cognition and behavior that usually exhibits a slow onset before worsening over time and ultimately leading to death. The causes of AD are poorly understood, although several etiological factors, such as genetic abnormalities, history of head injuries, environmental factors, general lifestyles, depression or hypertension, deposition of extracellular *ß*-amyloid protein (Aβ) and microtubule associated tau protein in the brain, and cholinergic dysfunction have all been implicated in AD. At present, there are no drugs available that are capable of curing Alzheimer’s disease or any of the other common types of dementia, but two conceptual approaches for the treatment of AD have been developed. Currently, only three cholinesterase inhibitors such as donepezil, galantamine and rivasigmine are the Food and Drug Administration (FDA) approved drugs to treat AD. Unfortunately, they only work for a short period of time, primarily in the early stages of the illness, to help patients delay the loss of cognitive functions as much as possible.

In this study, the HAp nanoparticles were selected as the drug delivery system with precise targeting. In general, nanoparticles are divided into two types: inorganic (metallic, metal oxide, and ceramic particles) and organic (organic, metal oxide, and ceramic particles) (lipidic and polymeric particles). The metallic NPs have some limitations over organic NPs because of the presence of metals, but few metallic NPs like gold, selenium and cerium NPs are reported to exhibit significant anti-AD properties. Recently in a study, the solid lipid NPs was shown to have significant inhibitory effects against amyloid aggregation Sathya et al. (2020). The selenium based NPs were seen to reduce the ROS level in the brain which is a key strategy to relieve AD because of the presence of many trace elements such as sodium selenite (VI), sodium selenite (IV) and selenium selenite (II) Fernandes and Gandin (2015). In the AD mouse model, the cerium NPs coupled with triphenylphosphonium (TPP) was seen to localize in the mitochondria to prevent the neuronal death Kwon et al. (2016). In another study, the gold NPs (AuNPs) showed significant results in reducing the symptoms of AD by modulating the mitochondrial functions dos Santos Tramontin et al. (2019).

When compared to the above mentioned nanoparticles AC-HAp NPs are not only bioactive but also non-toxic and non-immunogenic and do not contain any toxic elements Yasukawa et al. (1994). The AC-HAp NPs exhibit improved densification and better bioactivity than pure HAp NPs. None of the above discussed NPs were checked for their AChE inhibitory activity but in our present study, *A. calamus* rhizome with revitalizing neurological properties has been used to produce the HAp NPs and their AChE inhibitory activity was evaluated and has shown a promising AChE inhibitory action.

Inhibition of AChE, the key enzyme in the breakdown of acetylcholine, is considered one of the treatment strategies against Alzheimer’s disease. Plants have been traditionally used to enhance cognitive function and to alleviate other symptoms associated nowadays with Alzheimer’s disease. The AC-HAp NPs drastically enhanced the AChE inhibitory action even at very low concentrations when compared to pure HAp NPs. The IC50 values of 206.31 and 22.39 μg/ml were recorded with HAp NPs and AC-HAp NPs (Figure 6). Tacrine was used as a positive control which showed IC50 of 96.43 μg/ml. Surprisingly, significant AChE inhibition activity results was observed in *A. calamus* mediated HAp NPs, suggesting that the preparation of HAp NPs from *A. calamus* rhizome extract enhanced the AChE inhibition. Similar observations were made by Uddin et al. (2021) where the extracts of *Blumea lacera*, *Cyclea barbata*, *Smilax guianensis* and *Byttneria Pilosa* inhibited AChE with an IC50 values of 150 ± 11, 176 ± 14, 205 ± 31, and 221 ± 2 μg/ml respectively Uddin et al. (2021). They also proved that the plant extracts selected in their study showed a promising effect in inhibiting AChE activity, wherein the present study has shown improvement in biological activities in the medicinal plants when in combination with nanoparticle.

In the Molecular Docking studies, the strength of protein-ligand complex binding is well known as binding affinity. The affinity determines if the ligand binds to the target. Further, amongst the 20 phytocompounds screened, 5 compounds exhibited highest binding affinity and lowest binding energy values for the selected target proteins of AD, such as 1B8M, 1GS9, 3PFQ, 4L7G, and 5A63. The binding energy for all the target proteins was in the range of −5.7 to −10.7 kcal/mol with the formation of at least 6 to 11 hydrogen bonds. Depending upon the obtained binding energy, bonded and non-bonded interactions between the targets and 5 ligands (S-Adenosylhomocysteine, Carbenicillin, Apiin, Rutine and Chloramphenicol Monoglucuronide) the present study concludes that *A. calamus* phytocompounds have an effective anti-neurodegenarative activity.

## 5 Conclusion

*A. calamus* rhizome extract was used to successfully produce hydroxyapatite nanoparticles (AC-HAp NPs). The formation of nanoparticles was confirmed by SEM, EDX, XRD, TEM, HR-TEM and SAED techniques. The formation of AC-HAp NPs with high crystallinity and well defined forms was demonstrated by XRD, SEM, and TEM analysis. The goal of this study was to find phytoconstituents that can bind to the critical targets of amyloid hypothesis of AD using a computational approach and also to check the AChE inhibition activity of the synthesized AC-HAp NPs. The findings of the present study shows that as-prepared AC-HAp NPs can inhibit AChE, which was compared with pure AC-HAp NPs. *In silico* molecular docking approach revealed that most of the compounds derived from *A. calamus* rhizome extract have the ability to bind to the selected targets, according to the binding scores and analysis of the interactions of the compounds. Further, *in vivo* studies to evaluate substances like S-Adenosylhomocysteine, Carbenicillin, Apiin, Rutine and Chloramphenicol Monoglucuronide would lead to therapeutically effective molecules for treating a variety of chronic pain problems. It is also suggested that multipurpose NPs with multitherapeutic capabilities can be used. Given the present medications’ major targets of tau proteins, neuroinflammation, and Aβ proteins, there is an urgent need to create drugs with novel targets that can not only treat the symptoms but also prevent the disease from progressing at an early stage, resulting in a better life.

With the additional goal of analyzing their bioactivities, the predicted biological targets and the ADMET parameters related to the bioavailability and computational pharmacokinetics of the five ligands have been reported. The chemical reactivities of these five ligands have been thoroughly investigated through the optimization of their structures using the DFTBA methodology and the estimation of their electronic properties using the MN12SX/Def2TZVP/H2O model chemistry, which has already been used in previous research for the study of potentially therapeutic molecules, proving its suitability for this type of calculation and supporting this and previous research on this important subject.

## Data Availability

The original contributions presented in the study are included in the article/Supplementary Material, further inquiries can be directed to the corresponding authors.
