# Supplementary material for: Synthesis, Computational Pharmacokinetics Report, Conceptual DFT-Based Calculations and Anti-Acetylcholinesterase Activity of Hydroxyapatite Nanoparticles Derived From Acorus Calamus Plant Extract
Source: Front Chem. 2021 Oct 7;9:741037. doi: 10.3389/fchem.2021.741037 (PMC8529163; doi:10.3389/fchem.2021.741037)
Supplement: Supplementary file 1 [file DataSheet1.PDF]

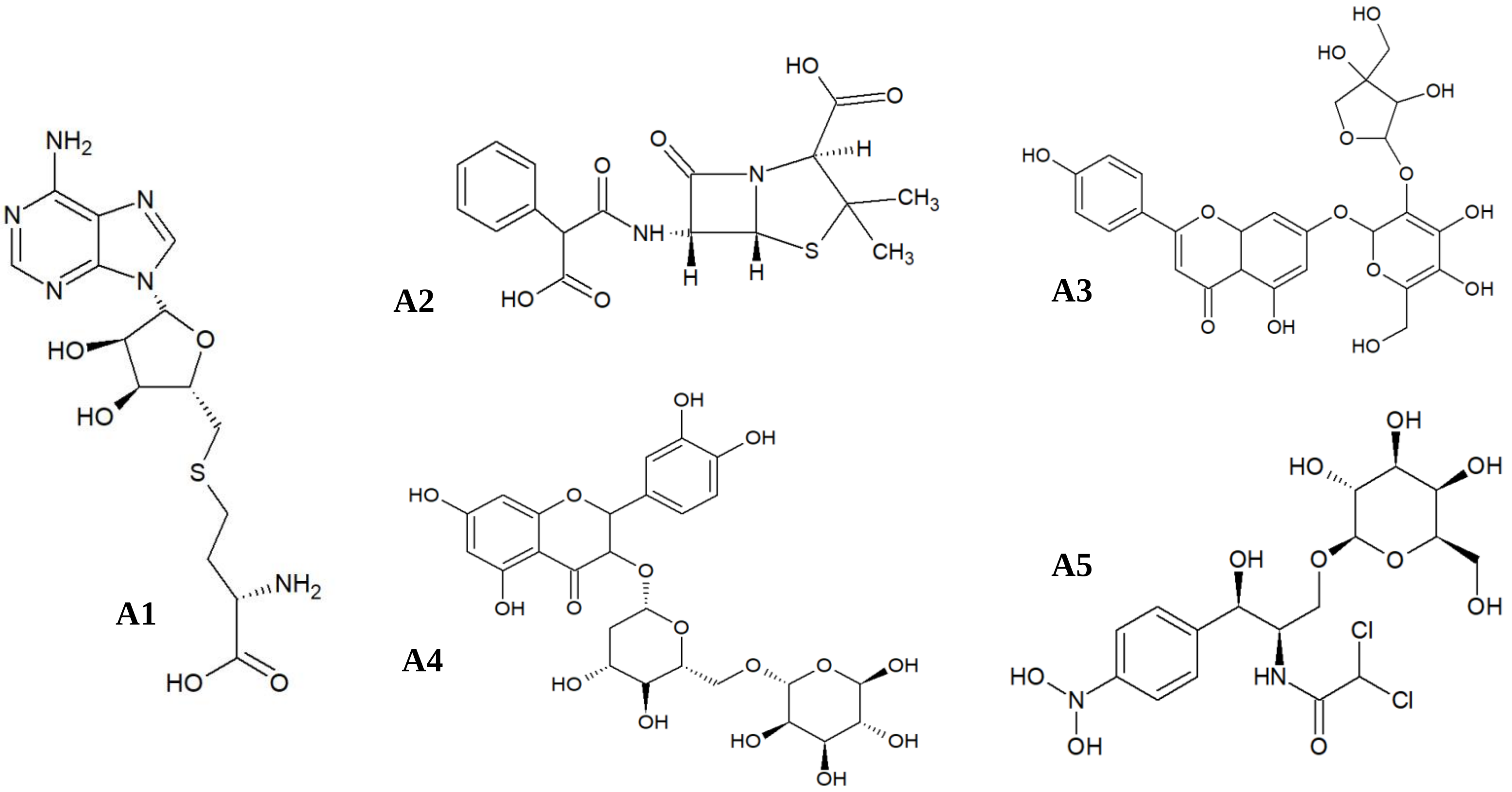

Figure S1: The 2D representation of the phytocompounds of *Acarus calamus* plant extract which showed good binding affinity towards the targets selected, where A1: Adenosylhomocysteine, A2: Carbenicillin, A3: Apiin, A4: Rutine and A5: Chloramphenicol Monoglucuronide

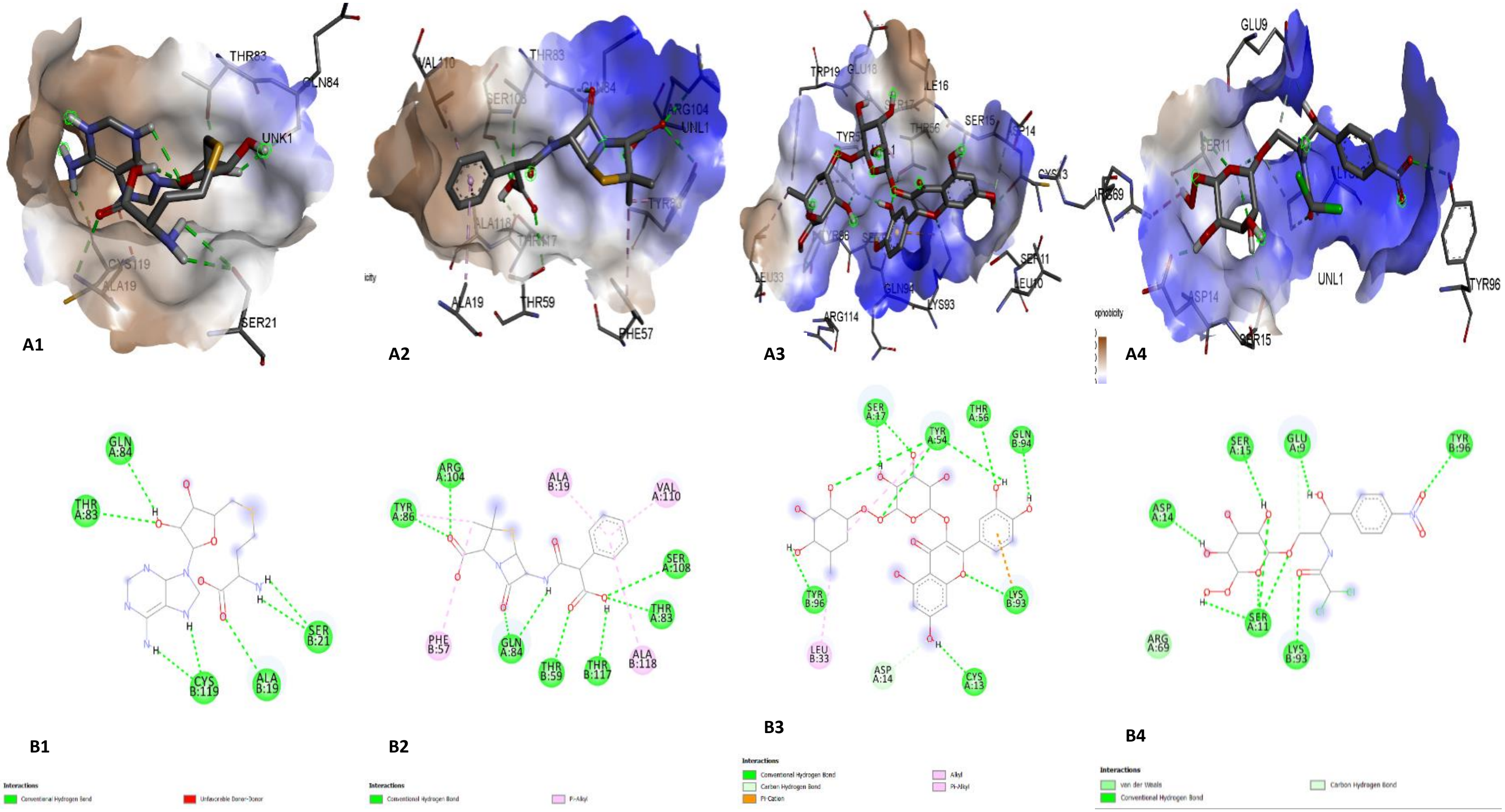

Figure S2. Molecular docking interaction analysis of protein 1B8M, A: 3D interactions and B: 2D interactions have been represented between the respective protein-ligand complex, where A1,B1: 1B8M-S- Adenosylhomocysteine, A2, B2: 1B8M-Carbenicillin, A3,B3: 1B8M-Rutine and A4,B4: 1B8M-Chloramphenicol Monoglucuronide complex structures

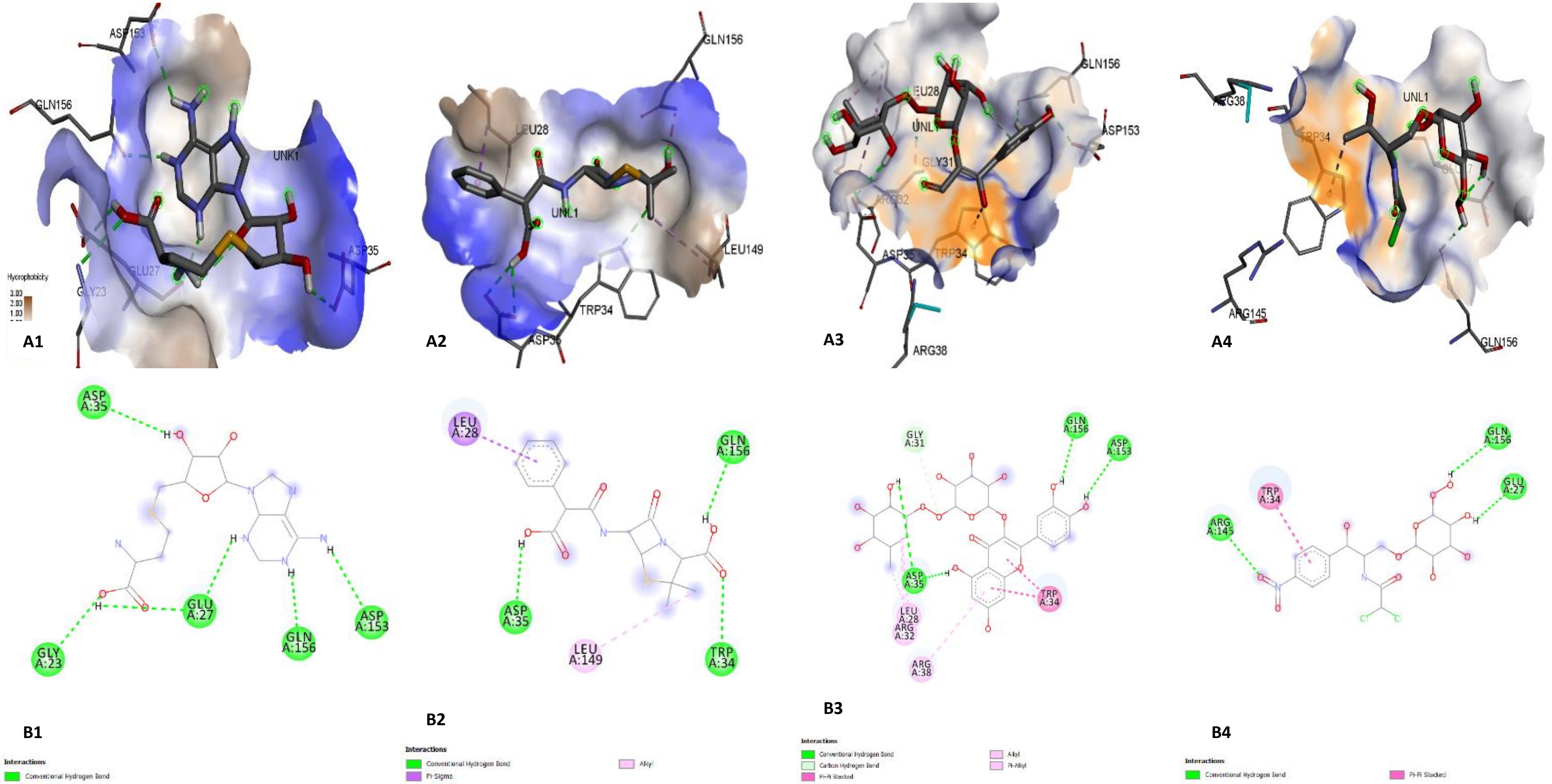

Figure S3. Molecular docking interaction analysis of protein 1GS9, A: 3D interactions and B: 2D interactions have been represented between the respective protein-ligand complex, where A1,B1: 1GS9- S-Adenosylhomocysteine, A2, B2: 1GS9-Carbenicillin, A3,B3: 1GS9-Rutine and A4,B4: 1GS9-Chloramphenicol Monoglucuronide complex structures

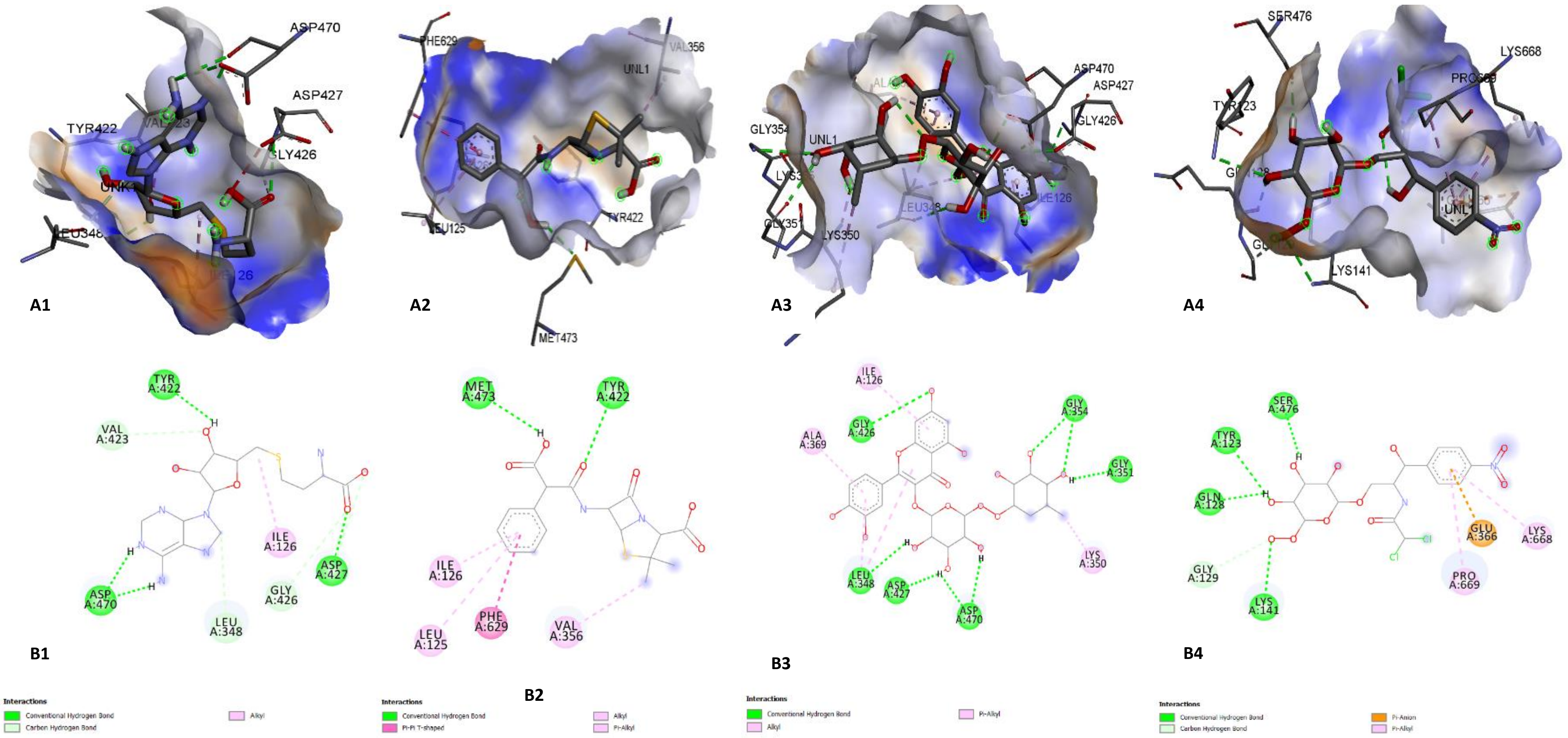

Figure S4. Molecular docking interaction analysis of protein 3PFQ, A: 3D interactions and B: 2D interactions have been represented between the respective protein-ligand complex, where A1,B1: 3PFQ- S-Adenosylhomocysteine, A2, B2: 3PFQ-Carbenicillin, A3,B3: 3PFQ-Rutine and A4,B4: 3PFQ-Chloramphenicol Monoglucuronide complex structures

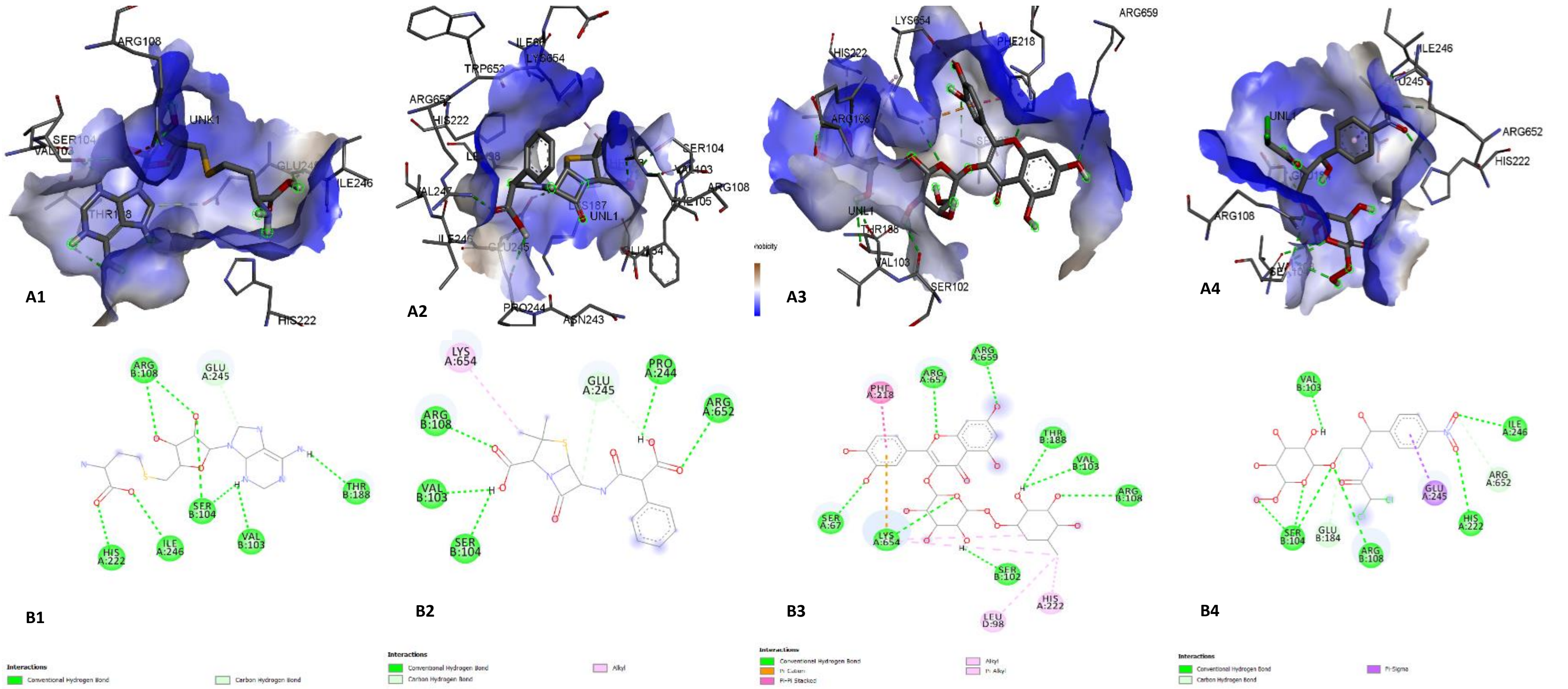

Figure S5. Molecular docking interaction analysis of protein 5A63, A: 3D interactions and B: 2D interactions have been represented between the respective protein-ligand complex, where A1,B1: 5A63- S-Adenosylhomocysteine, A2, B2: 5A63-Carbenicillin, A3,B3: 5A63-Rutine and A4,B4: 5A63-Chloramphenicol Monoglucuronide complex structures

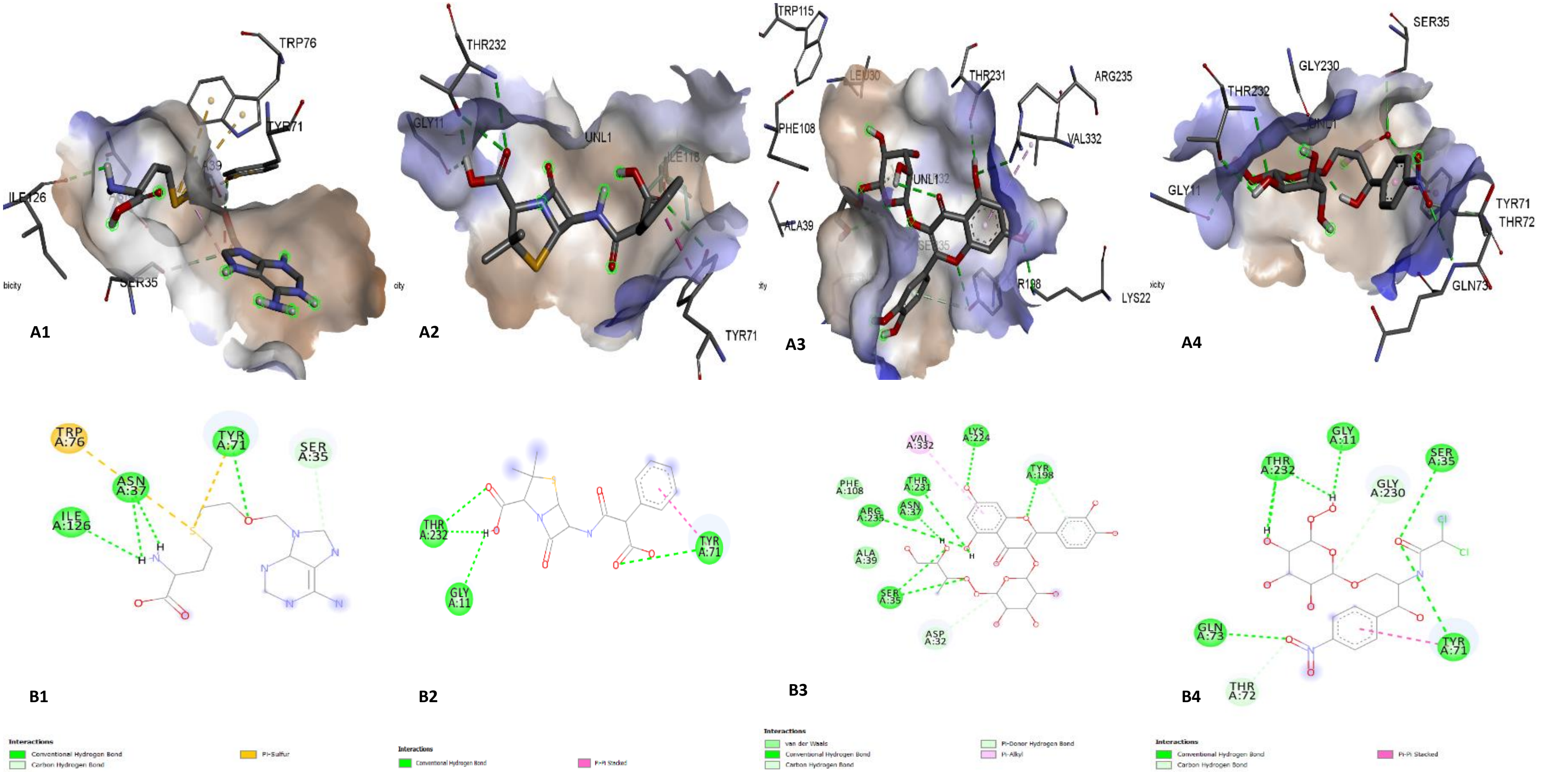

Figure S6. Molecular docking interaction analysis of protein 4L7G, A: 3D interactions and B: 2D interactions have been represented between the respective protein-ligand complex, where A1,B1: 4L7g- S-Adenosylhomocysteine, A2, B2: 4L7G-Carbenicillin, A3,B3: 4L7G-Rutine and A4,B4: 4L7G-Chloramphenicol Monoglucuronide complex structures.
